# Supplementary material for: Promoting multiexciton interactions in singlet fission and triplet fusion upconversion dendrimers
Source: Nat Commun. 2023 Sep 28;14:6080. doi: 10.1038/s41467-023-41818-1 (PMC10539328; doi:10.1038/s41467-023-41818-1)
Supplement: Supplementary file 1 — Supplementary Information [file 41467_2023_41818_MOESM1_ESM.pdf]

Supplementary Information

Promoting multiexciton interactions in singlet fission and triplet fusion upconversion dendrimers

Guiying He,[1,2] Emily M. Churchill,[3] Kaia R. Parenti,[3] Jocelyn Zhang,[3] Pournima Narayanan,[4,5] Faridah Namata,[6] Michael Malkoch,[6] Daniel N. Congreve,[4] Angelo Cacciuto,[3] Matthew Y. Sfeir,[1,2]* Luis M. Campos[3]*

[1]Department of Physics, Graduate Center, City University of New York, New York, NY 10016, USA

[2]Photonics Initiative, Advanced Science Research Center, City University of New York, New York, NY 10031, USA

[3]Department of Chemistry, Columbia University, New York, New York 10027, USA

[4]Department of Electrical Engineering, Stanford University, Stanford, California 94305, USA

[5]Department of Chemistry, Stanford University, Stanford, California 94305, USA

[6]KTH Royal Institute of Technology, Department of Fibre and Polymer Technology, SE-100 44 Stockholm, Sweden

* lcampos@columbia.edu

* msfeir@gc.cuny.edu

Table of Contents

I. Supplementary Discussion

A. Steady-state Spectra

B. Triplet-Triplet Upconversion Photoluminescence

C. Numerical Simulations

D. Transient Absorption Measurements

II. Supplementary Methods

A. Synthesis of PhBr Dendrimers

B. Synthesis of TIPS-Pentacene Dendrimers

C. Synthesis of TIPS-Anthracene Dendrimers

III. Supplementary Notes

A. MALDI Spectra

B. GPC Data

C. NMR Spectra

IV. Supplementary References

I. Supplementary Discussion

A. Steady-state Spectra

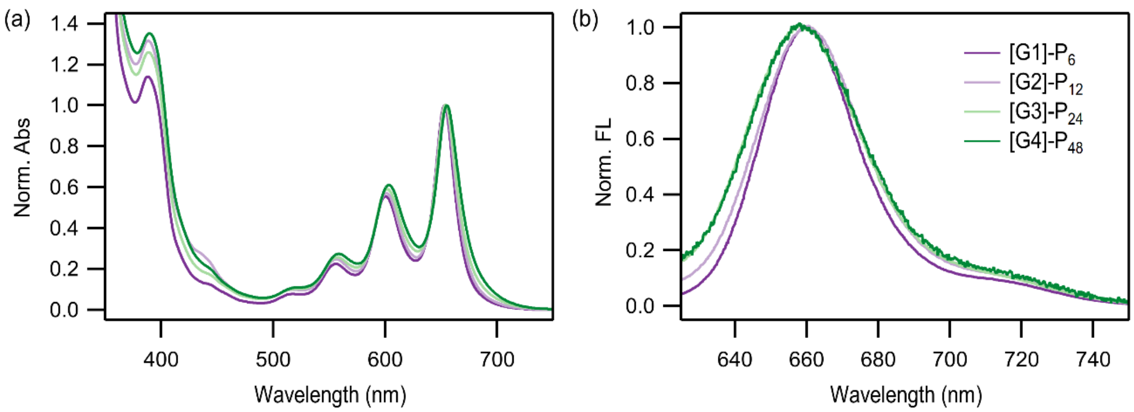

Supplementary Fig. 1. (a) Normalized absorption and (b) fluorescence spectra of the SF dendrimers [G1]-P_6, [G2]-P_{12}, [G3]-P_{24} and [G4]-P_{48}.

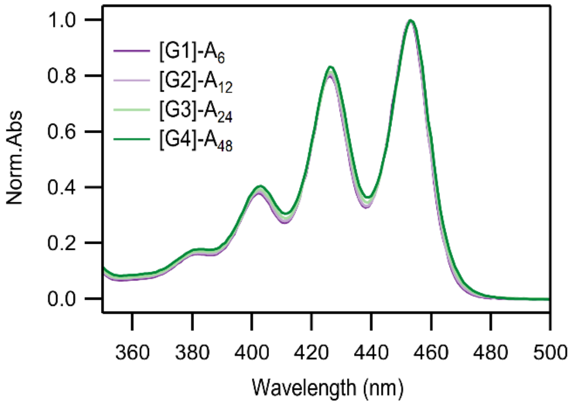

Supplementary Fig. 2. Normalized absorption spectra of anthracene dendrimers.

Supplementary Table 1. Photophysical properties of TIPS-An functionalized Bis-MPA dendrimers measured in 0.03 mg/mL in chloroform. Φ_{PL} measured at 40 μM effective annihilator concentration.

Compound	Abs λ_{max} (nm)	Em λ_{max} (nm)	ε $(Mcm)^{-1}$	Φ_{PL} (%)
[G1]-A_6	453	462	1.3 $x10^5$	53.8
[G2]-A_{12}	453	462	3.5 $x10^5$	62.2
[G3]-A_{24}	453	463	5.3 $x10^5$	59.8
[G4]-A_{48}	453	463	1.7 $x10^6$	49.5
TIPS-An-BE	452	461	4.1 $x10^4$	56.1

B. Triplet-Triplet Upconversion Photoluminescence

At 1 μM, TIPS-An-BE produced little UCPL signal. The dendrimers, however, produced measurable UCPL listed in Supplementary Table 2 and shown in Supplementary Figs. 3 and 4. All dendrimers had higher integrated UCPL intensity than TIPS-An-BE at comparable annihilator concentrations. The integrated UCPL of [G1]-A_6 and [G2]-A_{12} was 16× and 18× that of TIPS-An-BE, respectively. [G3]-A_{24} and [G4]-A_{48} had integrated UCPL of 5× and 2× that of TIPS-An-BE at comparable annihilator concentrations, respectively.

There is a noticeable shift in the UCPL spectra with increasing dendrimer size (Figure 2c, and normalized PL in Supplementary Fig. 3). The UCPL spectrum of [G1]-A_6 most similarly resembles that of TIPS-An-BE but with increased dendrimer generation there is a decrease in UCPL at the highest energy peak (462 nm) and increase UCPL at lower energy (500-600 nm). This is indicative of excimer formation, typically observed in environments which promote aggregation, such as concentrated solutions or in poorly solubilizing solvents. These results support that larger dendrimers increase the local annihilator concentration, even into a regime where TTA-UC becomes less efficient.

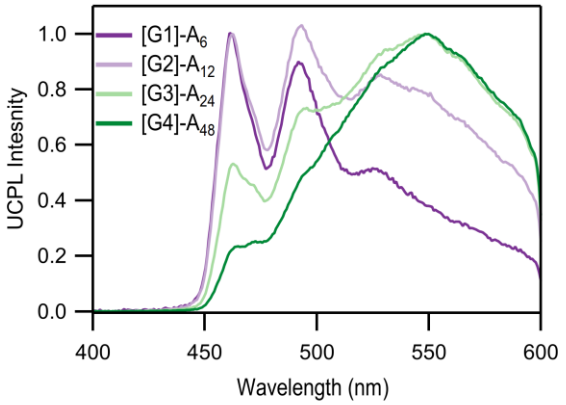

Supplementary Fig. 3. The normalized TTA-UCPL spectra of anthracene dendrimers.

Increasing the concentration of dendrimer in solution from 1 to 5, 10, 25, and 50 μM, with the concentration of PdTPTBP constant at 50 μM, results in an increasing intensity in the UCPL. Integrated UCPL intensity is summarized in Table 2. At low effective annihilator concentrations, dendrimers produce a higher UCPL intensity than TIPS-An-BE monomer, regardless of dendrimer

generation. Above 100 μM, efficiency of TIPS-An-BE increases such that its resulting UCPL intensity is higher than any of the studied dendrimers above 200 μM effective annihilator concentration. Compared to the integrated UCPL intensity of the other dendrimers, [G2]-A_{12} performs the best. Following [G2]-A_{12} are [G1]-A_6 and [G3]-A_{24}, which have similar UCPL intensities to each other at similar annihilator concentrations. Finally, [G4]-A_{48} performs the poorest at all studied effective annihilator concentrations. At low concentrations, it's favorable to have multiple annihilators locked into one core with proximity. However, there is a trade-off that maintaining too high of a local annihilator concentration using higher generation dendrimers results in the formation of excimers, which dominate the UCPL spectra. At higher concentrations, where intermolecular collisions are fast and efficient, freely diffusing monomers give yields that are comparable to the best dendrimer [G2]-A_{12} (Supplementary Fig. 4).

Supplementary Table 2. UCPL intensity integrated between 400-600 nm of [Gn]-A_x at dendrimer concentrations 1, 5, 10, 25, and 50 μM with 50 μM PdTPTBP in degassed chloroform. Reported as: intensity squared ± standard deviation calculated over triplicate measurements (effective annihilator concentration).

Dendrimer concentration (μM)	[G1]-A_6	[G2]-A_{12}	[G3]-A_{24}	[G4]-A_{48}
1	**24,700** ±600 (6 μM)	**59,000** ±1,000 (12 μM)	**114,000** ±6,000 (24 μM)	**148,000** ±6,000 (48 μM)
5	**235,000** ±5,000 (30 μM)	**839,000** ±6,000 (60 μM)	**980,000** ±40,000 (120 μM)	**990,000** ±20,000 (240 μM)
10	**490,000** ±30,000 (60 μM)	**1,500,000** ±30,000 (120 μM)	**1,670,000** ±80,000 (240 μM)	**1,480,000** ±50,000 (480 μM)
25	**1,220,000** ±30,000 (150 μM)	**2,880,000** ±20,000 (300 μM)	**2,900,000** ±100,000 (600 μM)	**2,240,000** ±60,000 (1200 μM)
50	**2,300,000** ±60,000 (300 μM)	**4,360,000** ±30,000 (600 μM)	**3,900,000** ±200,000 (1200 μM)	**2,230,000** ±70,000 (2400 μM)

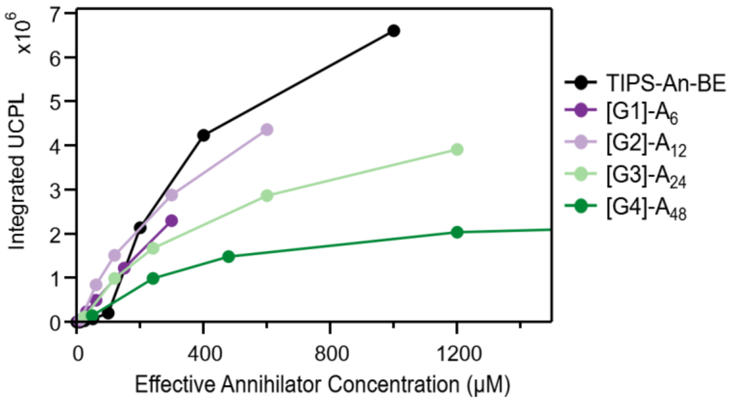

Supplementary Fig. 4. Integrated UCPL intensity as a function of annihilator concentration.

To quantify the yields of triplet energy transfer from sensitizer (PdTPTBP) to annihilator (monomer or dendrimer), we performed sensitization measurements with a fixed sensitizer concentration (~50 μM) using ns-TA. The decay dynamics of excited state of PdTPTBP (~510 nm) were measured to extract the yield of triplet transfer to the annihilators. At low annihilator concentrations of 1 μM, the triplet kinetics of PdTPTBP are identical to a neat solution. As the annihilator concentration is increased, the rate decay of the PdTPTBP triplet population increases. We measure a triplet transfer yield of 40% at ~50 μM annihilator concentration, corresponding to ~ 5% probability of transferring two triplet excitons to the same dendrimer. This value agrees well with UCPL characterization, which exhibits a low but measurable yield.

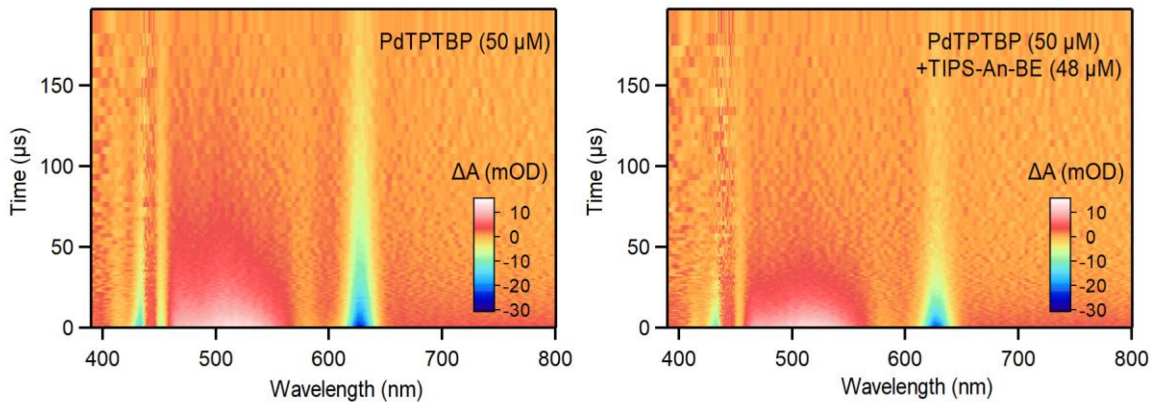

Supplementary Fig. 5. Sensitization measurements using ns-TA. PdTPTBP was excited by 635 nm.

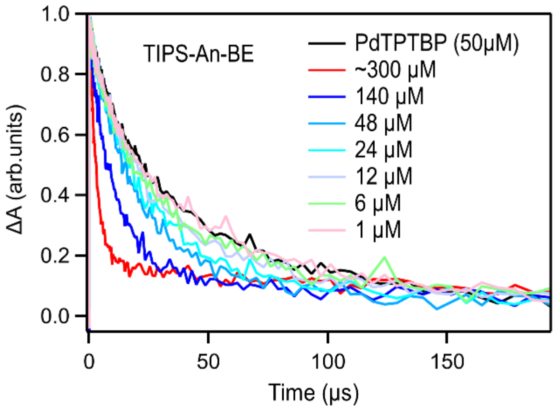

Supplementary Fig. 6. Triplet quenching dynamics of PdTPTBP with monomer annihilator (TIPS-An-BE) of different concentrations.

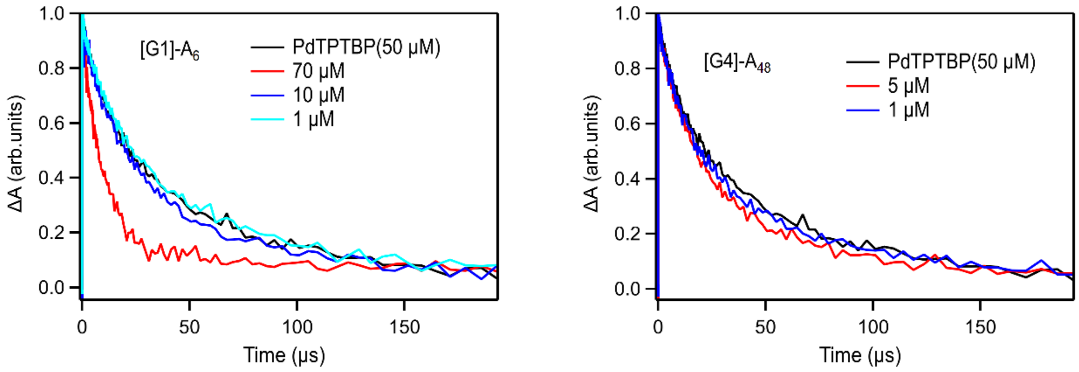

Supplementary Fig. 7. Triplet quenching dynamics of PdTPTBP with dendrimer annihilator ([G1]-A_6 and [G4]-A_{48}) of different concentrations.

C. Numerical Simulations

We modeled the dendrimer using a bead-and-spring model held together by harmonic springs of the form $U_s = \kappa/2\ (r-r_0)^2$ with spring constant $\kappa=300\ k_BT/\sigma^2$. Here σ is the diameter of the monomers, k_B is the Boltzmann constant, and the equilibrium distance $r_0=\sigma$. To enforce excluded volume interactions between the monomers, we use a MIE potential of the form $U_e= 4\varepsilon\,[\ (\sigma/r)^{24} - (\sigma/r)^{12}\ ]$, cut of at $r=2^{1/12}\sigma$, with $\varepsilon=1\ k_BT$.

The outer pentacene molecules are modeled as rectangular frames formed by a matrix of 3xδ spherical particles also of diameter σ (here δ is the longitudinal length of the pentacene molecule

which we vary) arranged in a planar square lattice as shown in Supplementary Fig. 8a. The shape of the pentacene is held together by the spring potential U_s and a bending potential of the form $U_b=k_b(\phi-\pi)^2$ between the particles along the columns and along the rows of the rectangle. The bending rigidity is set to k_b= 250 k_BT. The particles on the sides of the rectangle are there to merely enforce the planar shape of the molecule and interact with any other monomer in the dendrimer with the same excluded volume potential U_e. The monomers in the middle mediate the pentacene-pentacene attractive interactions which are set-up using the same MIE potential defined above to impose excluded volume interactions, but now with a cut-off that extends up to r=1.5σ (so that an attractive interaction emerges on top of the excluded volume) and ε in this case is set in the range of (3.5 – 5.5) k_BT (the strength of the attraction).

The molecular dynamics simulations are carried out using the numerical package LAMMPS[1] and the equations of motion follows a standard Langevin dynamics $\frac{d^2r}{dt^2}+g\frac{dr}{dt}=F+h$. Here g is the friction coefficient, which is set to 1, h is the Gaussian distributed and uncorrelated thermal noise, and F are the forces arising from the derivative of the potentials discussed above.

In our simulations σ and k_BT are used as the units of length and energy scales of the system, while $\tau=\sigma^2/D$ is our unit of time (D= k_BT/g is the diffusion coefficient of a monomer). All simulations were run for a minimum of 10^8 steps with time step Δt=$10^{-3}\tau$. Supplementary Fig. 8b shows the full model for a G2 dendrimer with δ=4.

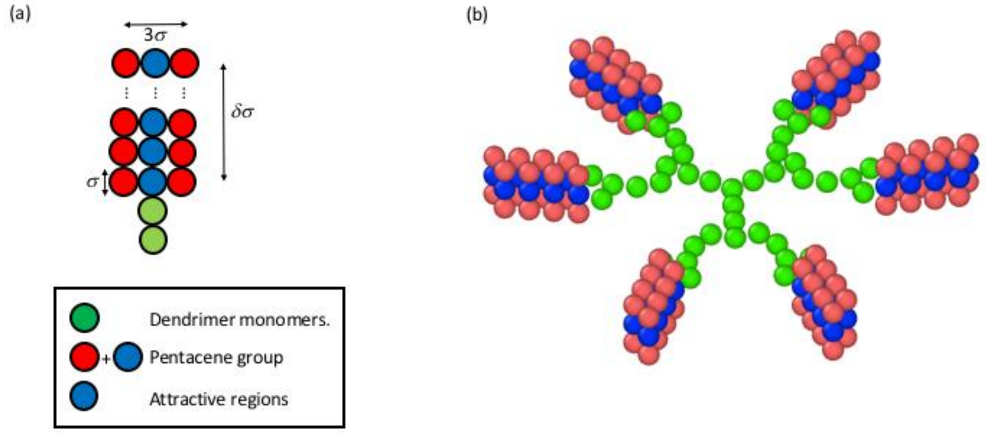

Supplementary Fig. 8. (a) Sketch of the Pentacene model. (b) Initial configuration from our simulations of a G2 dendrimer (δ=4).

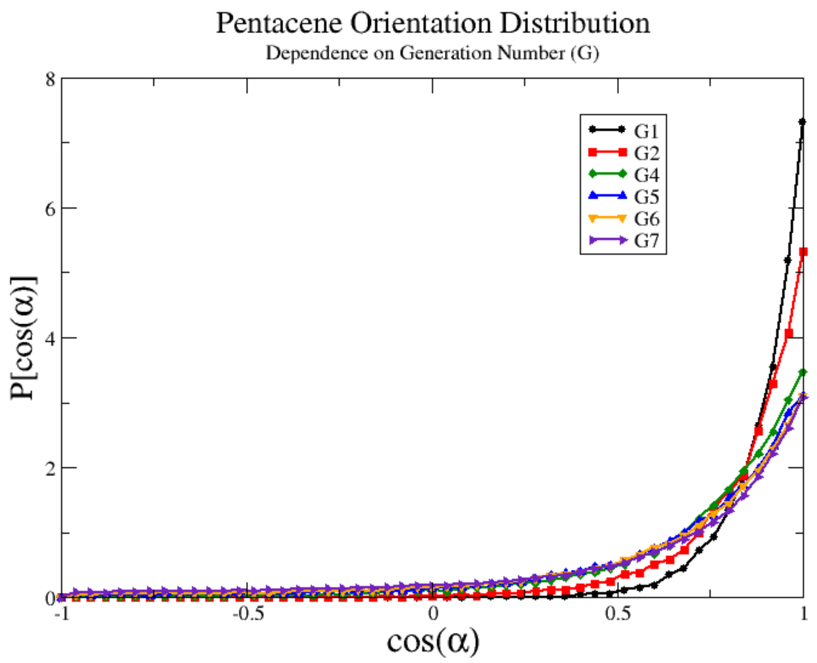

Supplementary Fig. 9. Probability distribution of cos(α) for dendrimers of different generation G. α is the angle formed between the unit vector, ***p***, along the direction connecting the center of the pentacene group to the center of the dendrimer, and the unit vector ***q***, parallel to the orientation of the pentacene group. This plot shows how increasing the generation number increases the pentacene orientation away from the center of the dendrimer. This data was collected for δ=4.

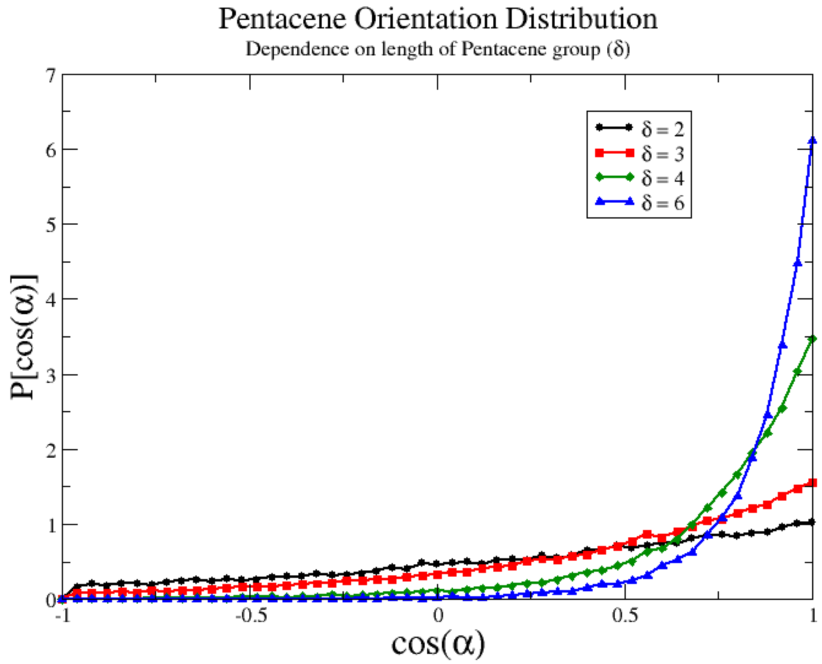

Supplementary Fig. 10. Probability distribution of cos(α) for dendrimers with pentacene groups of different length δ. α is the angle formed between the unit vector, **p**, along the direction connecting the center of the pentacene group to the center of the dendrimer, and the unit vector, **q**, parallel to the orientation of the pentacene group. Decreasing the length of the pentacene groups, δ, leads to less oriented pentacenes. This data corresponds to a G=4 dendrimer. For the smallest value of δ we observe a non-insignificant probability that the pentacenes fold back, i.e., reorient to point towards the center of the dendrimer.

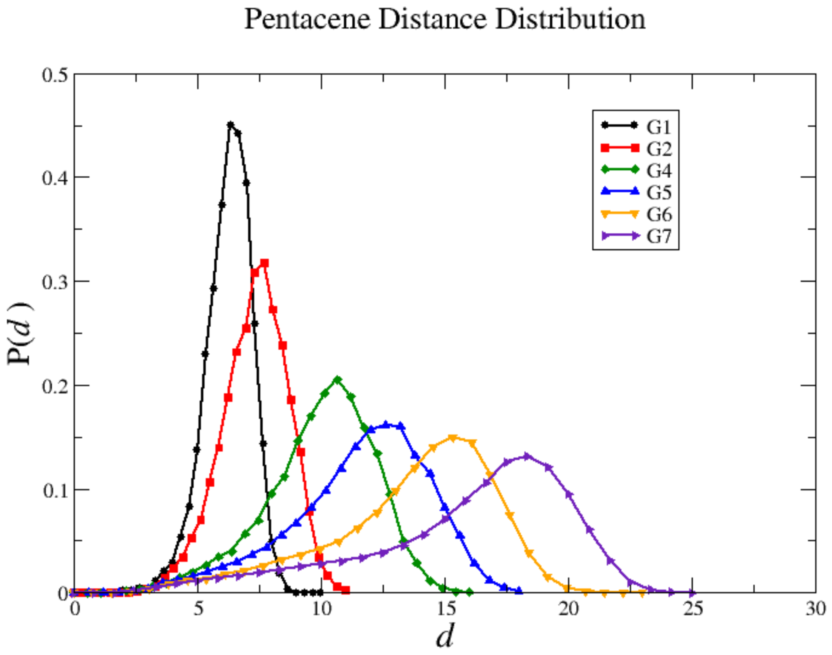

Supplementary Fig. 11. Probability distribution of the radial distance of the center of mass of the pentacene groups, *d*, from the center of the dendrimer for different generation numbers G1-G7, and fixed δ=4. Increasing the generation number increases the density of dendrimer monomers on its outer shells causing some of the pentacene groups to move towards the inner core of the dendrimer to relax its mechanical stress. This is visible from the long tails that develop at small values of *d* in these probability distributions as one increases the generation number G.

D. Transient Absorption Measurements

a. Nanosecond TA of dendrimers

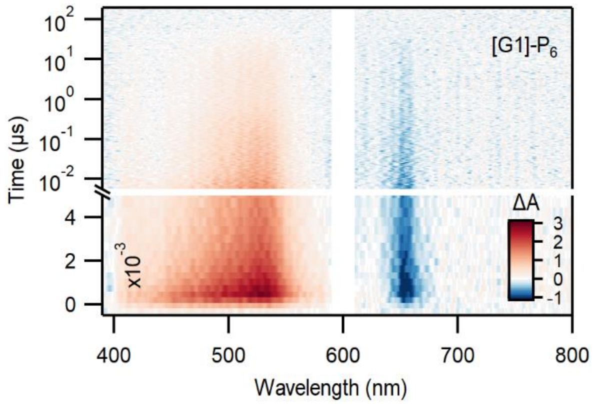

Supplementary Fig. 12. Nanosecond TA data of [G1]-P_6.

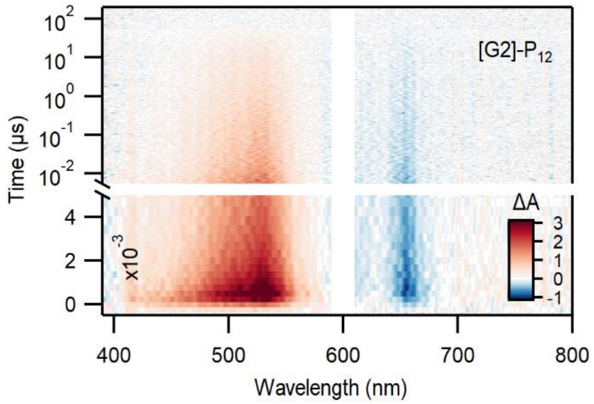

Supplementary Fig. 13. Nanosecond TA data of [G2]-P_{12}.

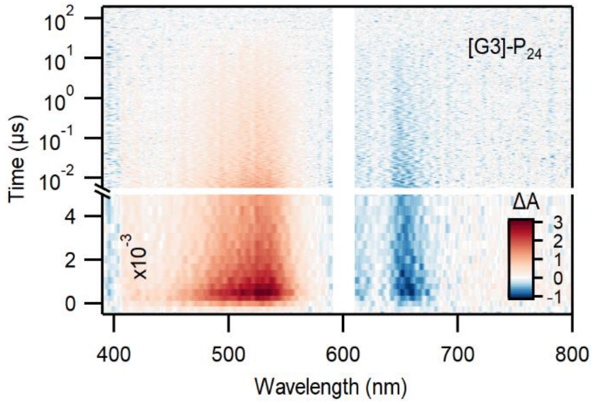

Supplementary Fig. 14. Nanosecond TA data of [G3]-P_{24}.

b. Target analysis of fs TA and global analysis of ns TA

To capture the full multiscale exciton dynamics of the SF dendrimers, we have constructed a model that partitions the spin conversion process into two distinct branches to account for the differing morphology of chromophores within a single dendrimer generation. For the fs TA data, our model starts with a common singlet state for Type I and Type II that then branches into two distinct ensembles. For simplicity, the branching ratio was set to 0.4 Type I and 0.6 Type II based on the approximate excited state population remaining following the initial fast (~ 1 ns) population loss (Figure 4e). Within the Type I branch, a portion of the initial singlet exciton population decays via a fast singlet fission step ($k_{SF,A}$) into a second species that represents a mixture of Type I specific singlet excitons and Type I triplet pairs. This is followed by a slower SF step ($k_{SF,B}$) that fully converts the excited state population to triplet pairs. The triplet pairs decay in the usual via exciton-exciton annihilation (k_{TT}) which leaves behind a residual population of free triplets that decays to the ground state (k_{T_1}). The overall process can be summarized as: $S_1 \xrightarrow{k_{SF,A}} S_1^I + TT^I \xrightarrow{k_{SF,B}} TT^I \xrightarrow{k_{TT}} T_1 \xrightarrow{k_{T_1}} G$. For Type II, a simpler scheme is used in which one characteristic rate constant describes the SF process ($k_{SF,A}$) and in which triplet pairs decay directly to the ground state (k'_{TT}): $S_1 \xrightarrow{k_{SF,A}} TT^{II} \xrightarrow{k'_{TT}} G$.

For the nanosecond TA data, a simpler sequential global analysis is used to extract time constants and spectra. The effectiveness of our model was evaluated by comparing the fitted spectra to the known singlet and triplet transient spectra, e.g., using triplet sensitization.

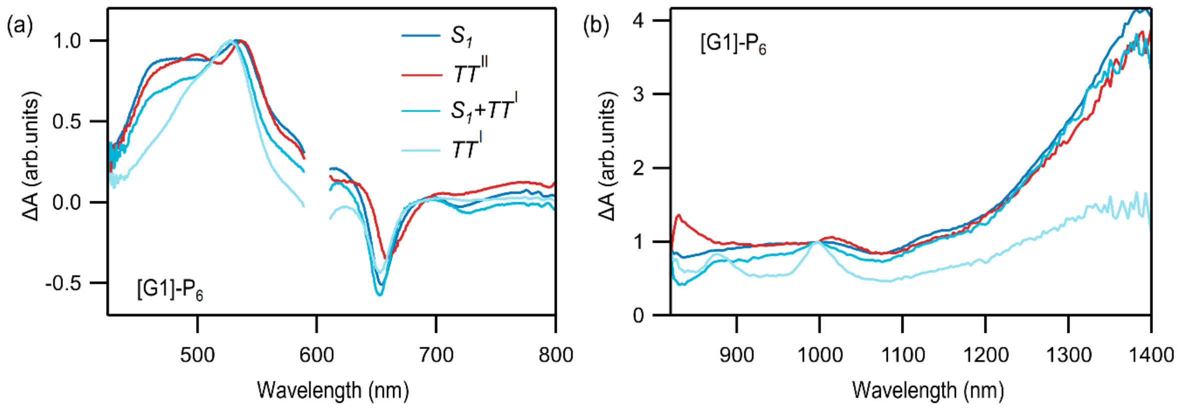

Supplementary Fig. 15. Target analysis of [G1]-P_6 visible (a) and NIR (b) fs-TA data.

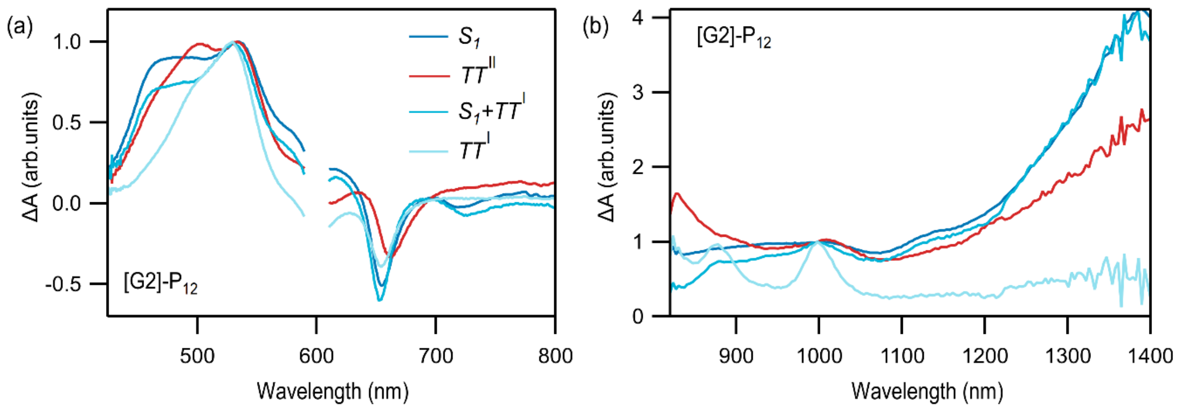

Supplementary Fig. 16. Target analysis of [G2]-P_{12} visible (a) and NIR (b) fs-TA data.

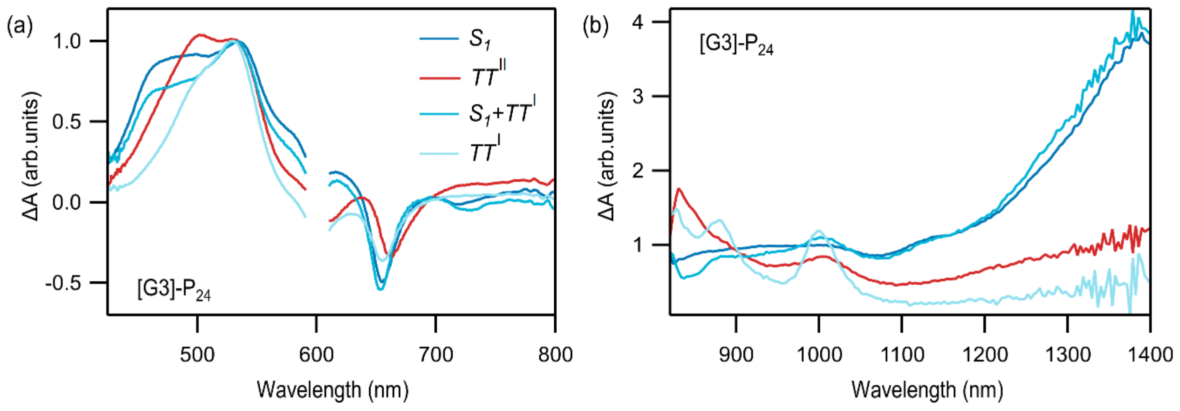

Supplementary Fig. 17. Target analysis of [G3]-P_{24} visible (a) and NIR (b) fs-TA data.

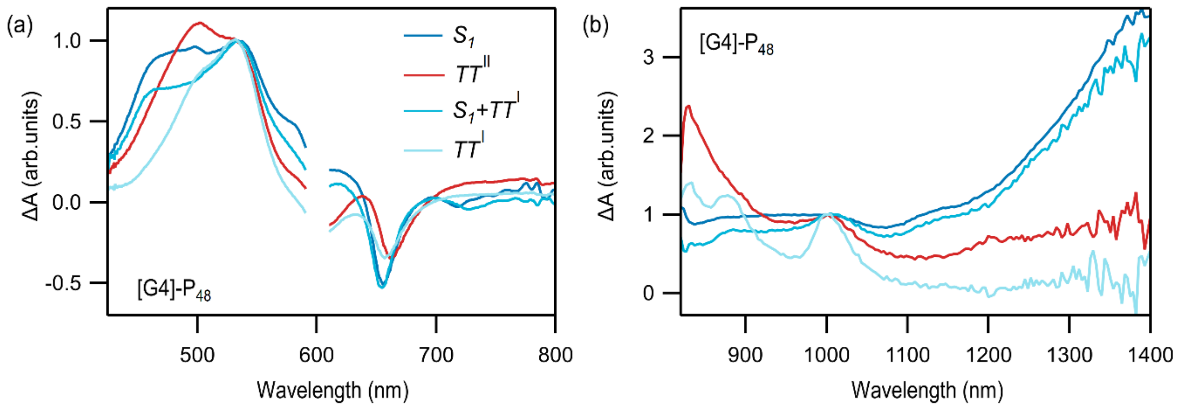

Supplementary Fig. 18. Target analysis of [G4]-P_{48} visible (a) and NIR (b) fs-TA data.

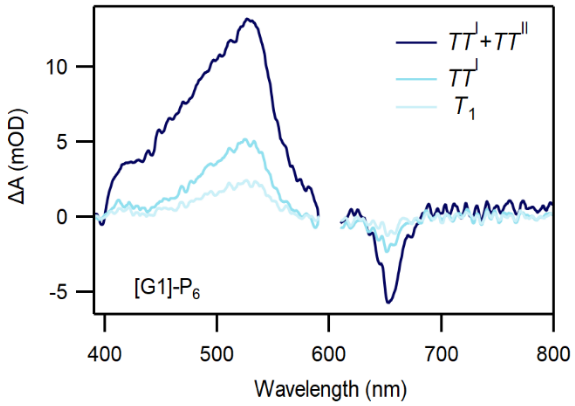

Supplementary Fig. 19. Target analysis of [G1]-P_6 visible ns-TA data.

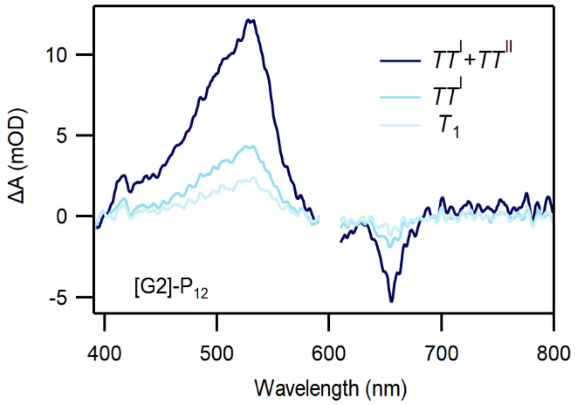

Supplementary Fig. 20. Target analysis of [G2]-P_{12} visible ns-TA data.

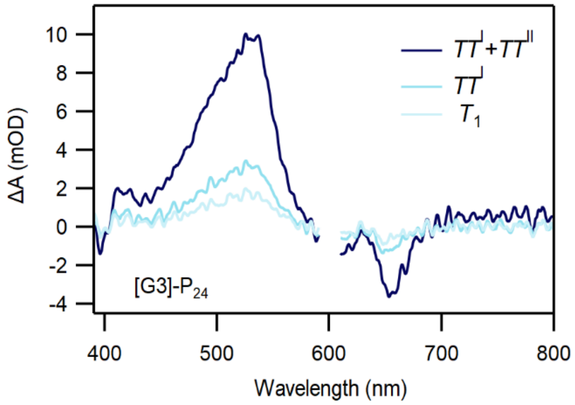

Supplementary Fig. 21. Target analysis of [G3]-P_{24} visible ns-TA data.

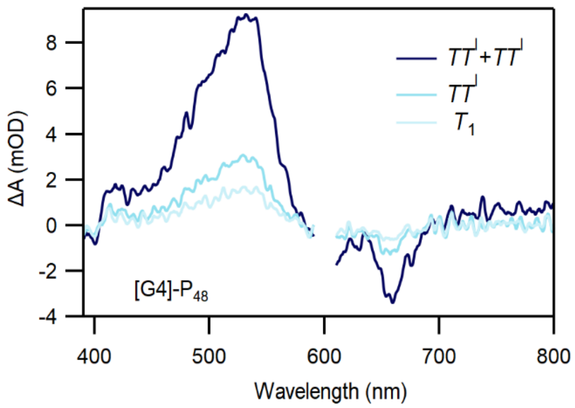

Supplementary Fig. 22. Target analysis of [G4]-P_{48} visible ns-TA data.

c. Photosensitization

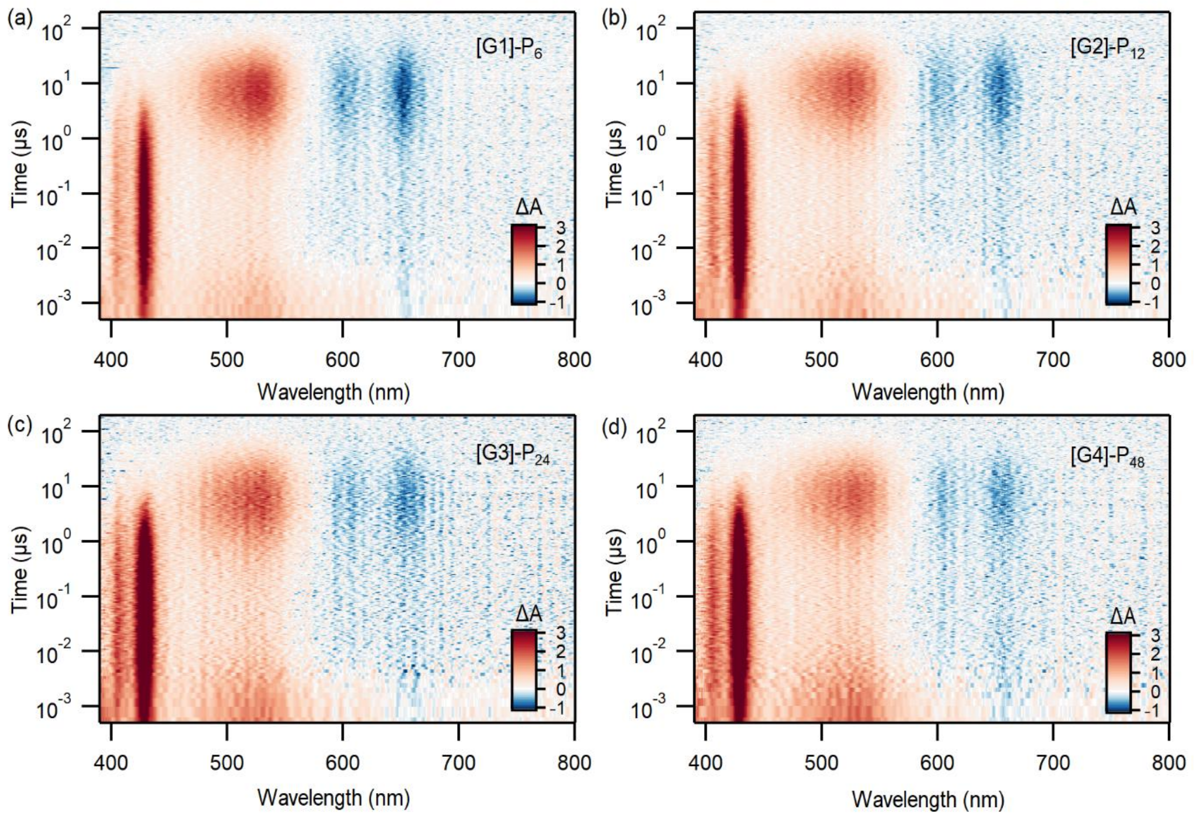

Supplementary Fig. 23. Triplet sensitization data of (a) [G1]-P_6, (b) [G2]-P_{12}, (c) [G3]-P_{24}, and (d) [G4]-P_{48}, where the excited state absorption near 420 nm corresponds to anthracene triplet state transition. The decay of that signal corresponds primarily with transfer to the rise of the pentacene dendrimer triplet signal.

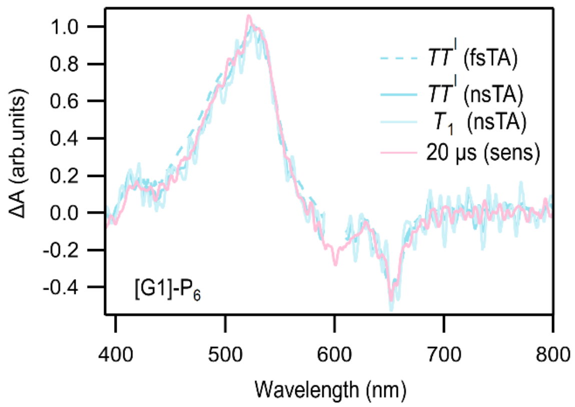

Supplementary Fig. 24. Spectral comparison of type I triplet pair state from fs-TA data and corresponding type I triplet pair state individual triplet state from ns-TA data and the sensitization triplet state of [G1]-P_6.

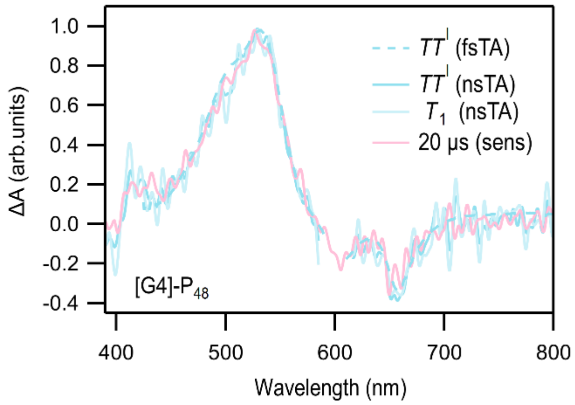

Supplementary Fig. 25. Spectral comparison of type I triplet pair state from fs-TA data and corresponding type I triplet pair state individual triplet state from ns-TA data and the sensitization triplet state of [G4]-P_{48}.

d. Fluence-dependent triplet decay measurements

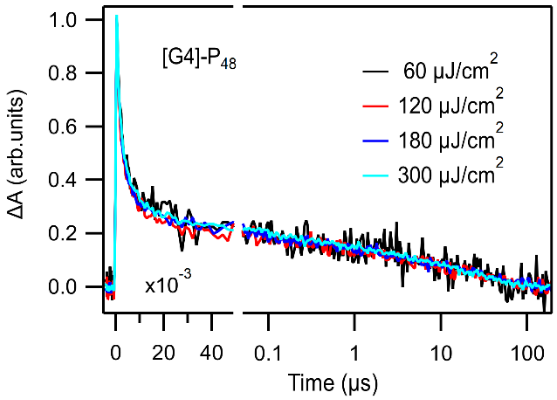

Supplementary Fig. 26. Fluence dependent ns-TA measurements for [G4]-P_{48}.

II. Supplementary Methods

A. Synthesis of PhBr Dendrimers

Adapted from a related literature procedure.[2] To a suspension of CDI (10 equiv. for G1/20 equiv. for G2/36 equiv. for G3/54 equiv. for G4) in EtOAc at 50°C was added 4-bromobenzoic acid (10 equiv. for G1/20 equiv. for G2/36 equiv. for G3/54 equiv. for G3). After stirring for 1 h at 50°C, CsF (1.2 equiv. for G1/2.4 equiv. for G2/4.8 equiv. for G3/9.6 equiv. for G4) and the hydroxyl-terminated dendrimer (1 equiv.) were added. The reaction was stirred at 50°C overnight. The reaction was cooled to r.t. and quenched by stirring with water for 1 h. After quenching, the mixture was diluted with EtOAc, washed with $NaHCO_3$, dried over Na_2SO_4, and concentrated down. The crude was dissolved in a minimal amount of EtOAc and purified by MeOH precipitation. A representative reaction is shown in Supplementary Fig. 27.

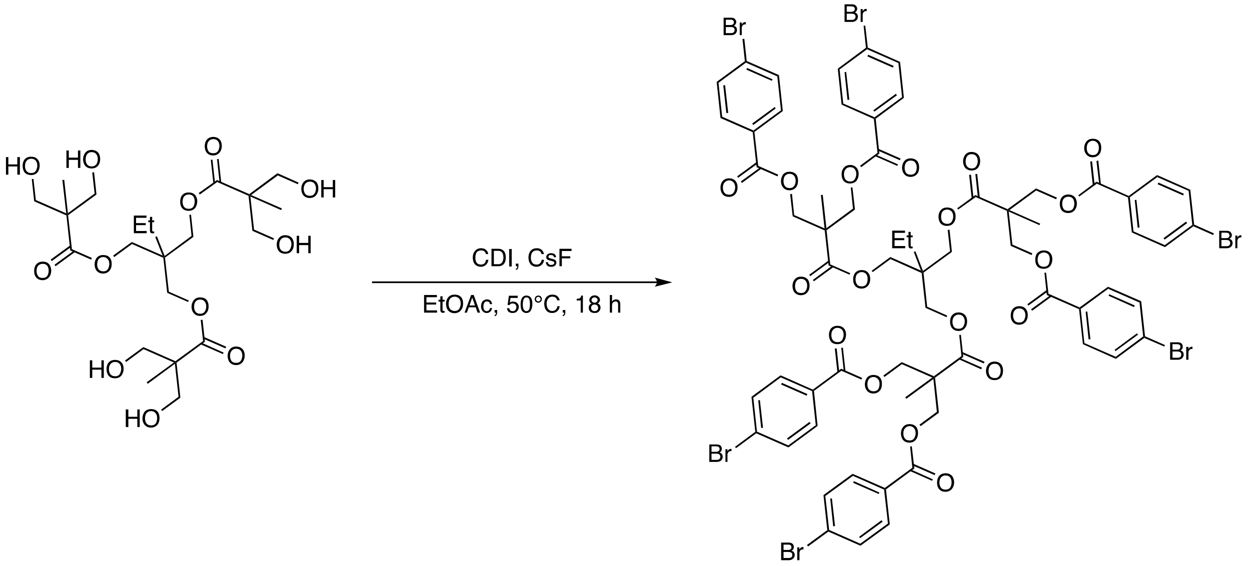

Supplementary Fig. 27. Synthesis of [G1]-Ph_6.

[G1]-Ph_6

Dendrimer [G1]-Ph_6 was obtained as a white solid after purification (0.207 mmol scale, 90% yield).

^{1}H-NMR (300 MHz, $CDCl_3$, δ): 7.81 (d, 12H), 7.55 (d, 12H), 4.55 – 4.45 (m, 12H), 4.04 (s, 6H), 1.34 (s, 11H), 0.66 (t, 3H).
^{13}C-NMR (126 MHz, $CDCl_3$, δ): 172.06, 165.27, 132.06, 131.24, 128.74, 128.40, 66.09, 63.77, 47.04, 41.73, 22.83, 18.12, 7.28.

[G2]-Ph_{12}

Dendrimer [G2]-Ph_{12} was obtained as a white solid after purification (0.085 mmol scale, 66% yield).

^{1}H-NMR (400 MHz, $CDCl_3$, δ): 7.81 – 7.76 (d, 24H), 7.54 – 7.50 (d, 24H), 4.52 – 4.44 (m, 24H), 4.33 – 4.21 (m, 12H), 3.92 (s, 6H), 1.34 (s, 21H), 1.17 (s, 9H), 0.76 (t, 3H).
^{13}C-NMR (126 MHz, $CDCl_3$, δ): 171.93, 171.59, 165.25, 132.01, 131.25, 128.67, 128.44, 66.06, 65.47, 64.09, 46.92, 18.07, 17.62, 7.52.

[G3]-Ph_{24}

Dendrimer [G3]-Ph_{24} was obtained as a white solid after purification (0.038 mmol scale, 82% yield).

^{1}H-NMR (300 MHz, $CDCl_3$, δ): 7.76 (d, 48H), 7.49 (d, 48H), 4.54 – 4.39 (m, 48H), 4.32 – 4.18 (m, 24H), 4.15 – 4.08 (m, 12H), 4.01 (s, 6H), 1.32 (s, 36H), 1.26 (s, 9H), 1.15 (s, 23H).
^{13}C-NMR (126 MHz, $CDCl_3$, δ): 171.94, 165.21, 132.00, 131.24, 128.64, 128.46, 66.04, 65.37, 46.91, 46.77, 18.04, 17.64, 17.56.

[G4]-Ph_{48}

Dendrimer [G4]-Ph_{48} was obtained as a white solid after purification (0.019 mmol scale, 71% yield).

^{1}H-NMR (300 MHz, $CDCl_3$, δ): 7.73 (d, 96H), 7.45 (d, 96H), 4.53 – 4.37 (m, 96H), 4.34 – 4.04 (m, 90H), 1.29 (s, 72H), 1.20 (s, 14H), 1.13 (m, 54H).
^{13}C-NMR (126 MHz, $CDCl_3$, δ): 171.92, 171.49, 171.40, 165.14, 131.96, 131.21, 128.58, 128.44, 66.01, 65.25, 46.87, 46.70, 46.54, 18.00, 17.65, 17.56.

B. Synthesis of TIPS-Pentacene Dendrimers

To a reaction vial was added PhBr dendrimer ([Gn]-$PhBr_x$, 1 equiv.), BPin TIPS Pc (10 equiv. for G1/16 equiv. for G2/30 equiv. for G3/54 equiv. for G4), $Pd(dppf)_2Cl_2$·DCM (1 equiv.), and K_2CO_3 (20 equiv. for G1/32 equiv. for G2/60 equiv. for G3/108 equiv. for G4). Three evacuate-refill cycles were performed to degas the mixture followed by the addition of a 5:1 mixture of dry THF and degassed H_2O (0.01M). The reaction was stirred at 75°C for 24 h in the dark. The reaction was cooled to room temperature, dried over Na_2SO_4, and concentrated down. The crude was purified by silica chromatography using a mixture of hexanes, DCM, and MeOH as the eluent. A representative reaction is shown in Supplementary Fig. 28.

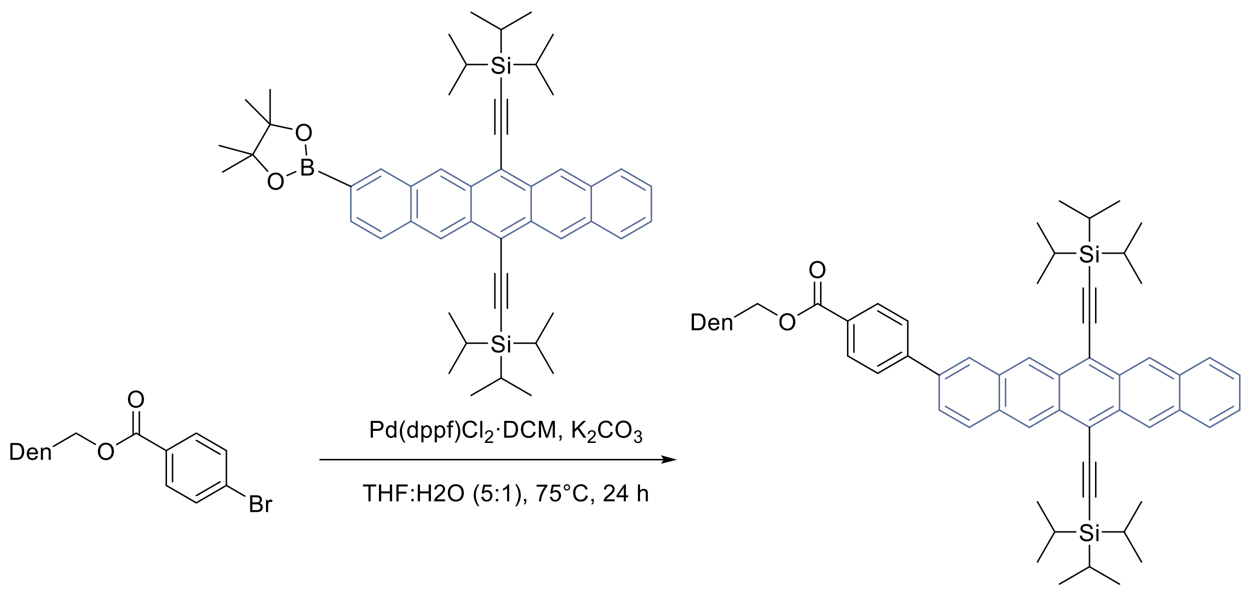

Supplementary Fig. 28. Synthesis of pentacene dendrimer.

[G1]-P_6

Dendrimer [G1]-P_6 was obtained as a teal solid after purification (0.02 mmol scale, 36% yield).

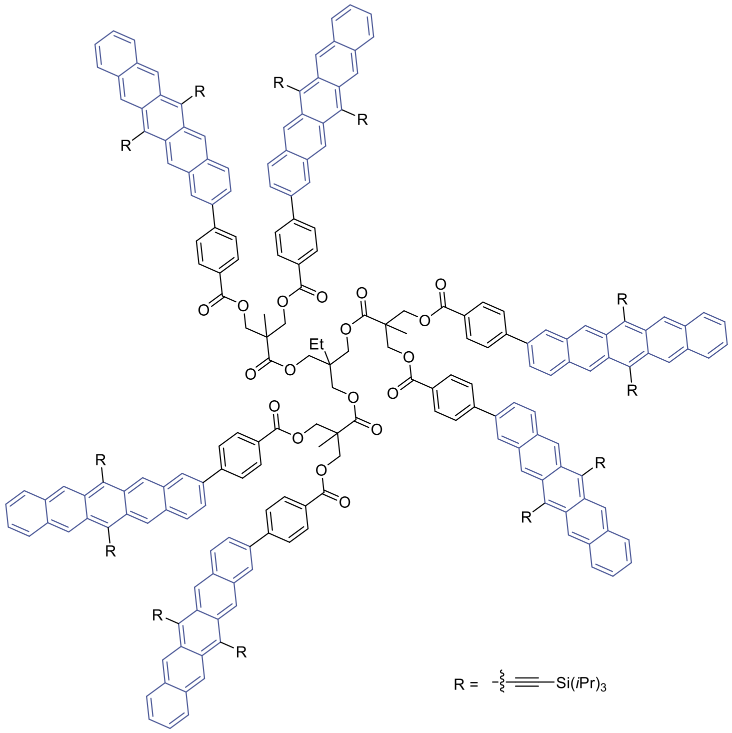

^{1}H-NMR (500 MHz, $CDCl_3$, δ): 9.26 – 9.10 (m, 24H), 8.08 (d, 12H), 8.01 (s, 6H), 7.94 – 7.83 (m, 18H), 7.75 (d, 11H), 7.52 (d, 6H), 7.28 (m, 13H), 4.65 (d, 6H), 4.53 (d, 6H), 4.23 (s, 6H), 1.35 (m, 266H).

^{13}C-NMR (126 MHz, $CDCl_3$, δ): 165.80, 145.31, 136.59, 132.37, 132.02, 131.45, 130.87, 130.75, 130.71, 130.36, 129.70, 128.68, 128.44, 127.15, 126.70, 126.37, 126.21, 126.10, 125.46, 118.55, 118.46, 107.44, 107.26, 104.75, 65.93, 29.86, 19.15, 19.13, 18.19, 11.81, 11.58.

[G2]-P_{12}

Dendrimer [G2]-P_{12} was obtained as a teal solid after purification (0.009 mmol scale, 46% yield).

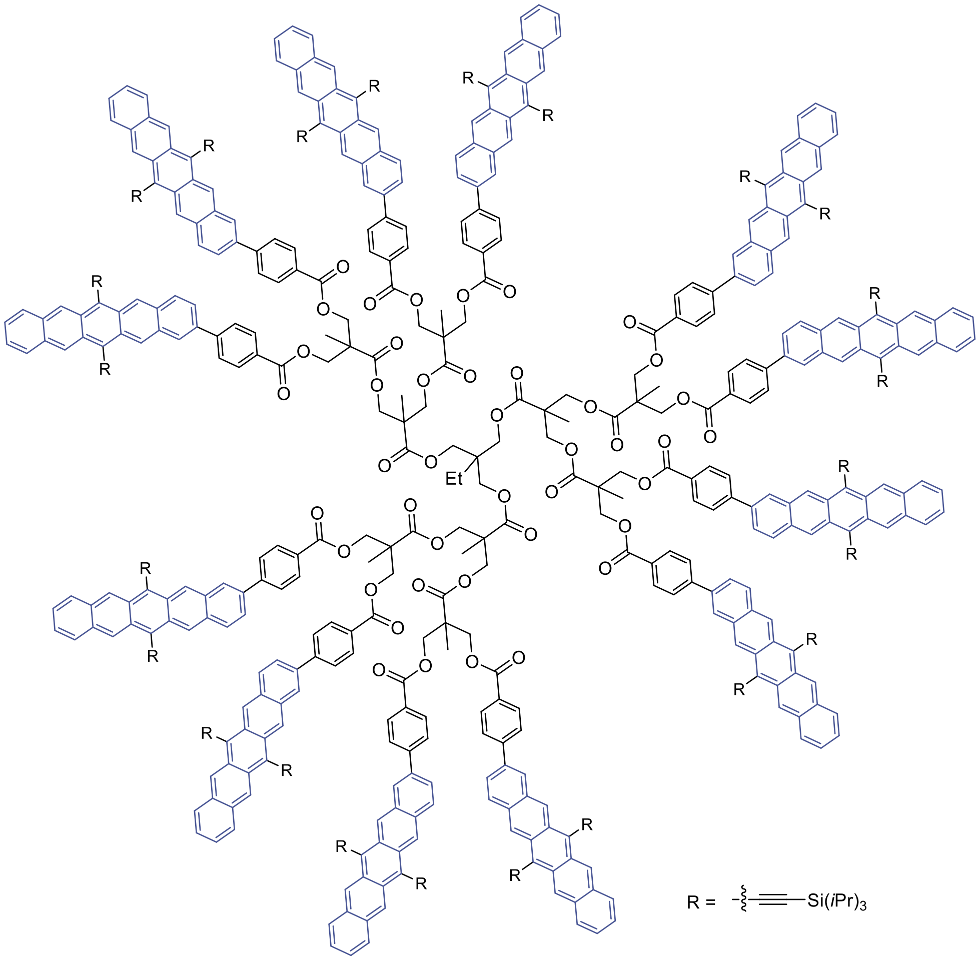

^{1}H-NMR (400 MHz, $CDCl_3$, δ): 9.18 – 9.04 (m, 44H), 8.09 – 8.00 (m, 25H), 7.94 (m, 13H), 7.81 (m, 35H), 7.68 (m, 22H), 7.57 – 7.40 (m, 17H), 7.22 (m, 24H, overlapping chloroform peak), 4.64 (d,12H), 4.58 – 4.40 (m, 24H), 4.22 (s, 6H), 1.29 (m, 536H).

[G3]-P_{24}

Dendrimer [G3]-P_{24} was obtained as a teal solid after purification (0.007 mmol scale, 12% yield).

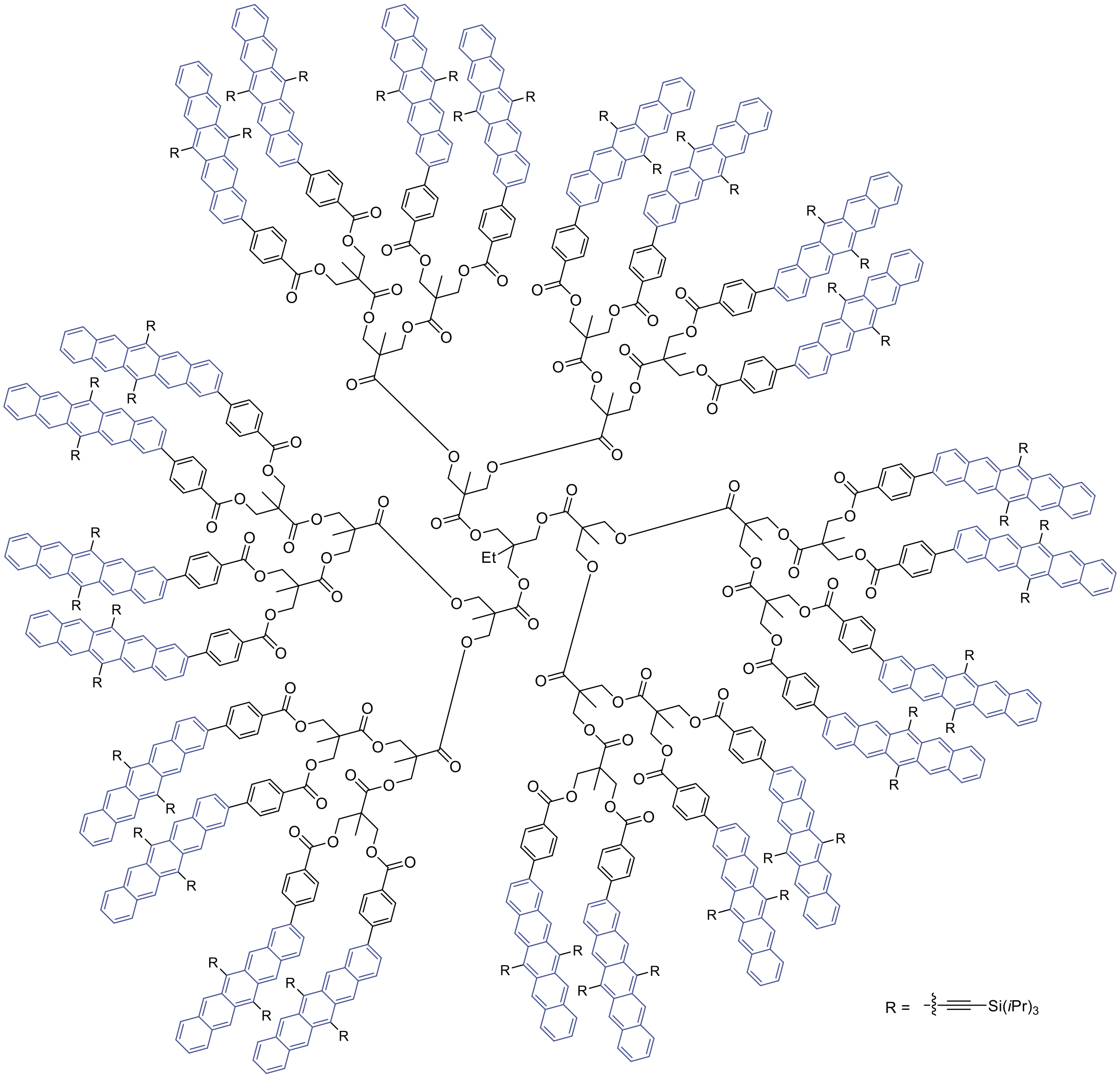

^{1}H-NMR (400 MHz, $CDCl_3$, δ): 9.12 – 8.90 (m, 90H), 8.06 – 7.57 (m, 211H), 7.37 (s, 25H), 7.14 (s, 40H), 4.75 – 4.41 (m, 90H), 1.30 – 1.13 (m, 1076H).

Limited solubility prevented acquisition of a ^{13}C NMR.

[G4]-P_{48}

Dendrimer [G4]-P_{48} was obtained as a teal solid after purification (0.002 mmol scale, 15% yield).

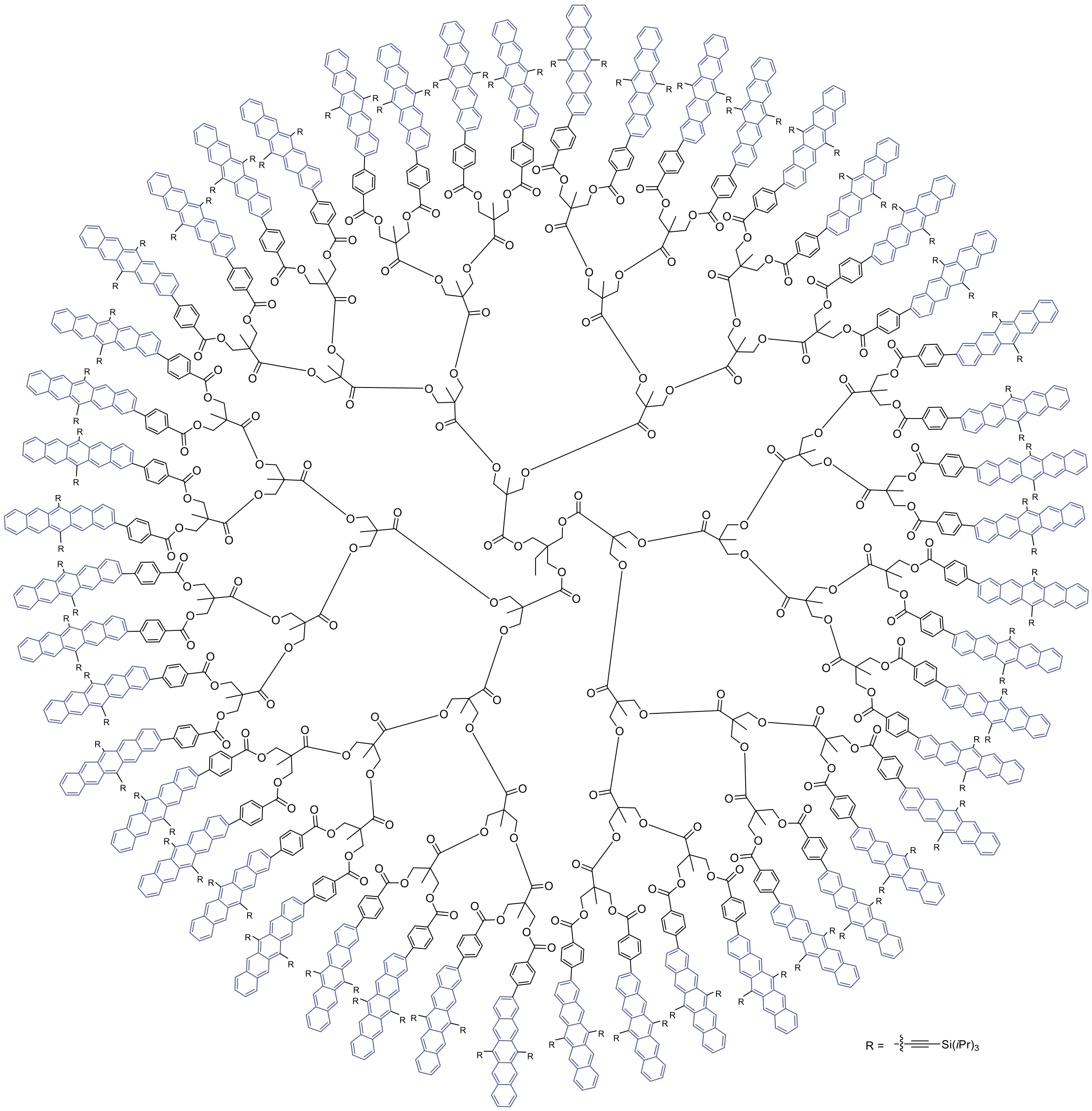

^{1}H-NMR (300 MHz, $CDCl_3$, δ): 9.08 – 8.67 (m, 188H), 7.92 (m, 117H), 7.80 – 7.39 (m, 296H), 7.23 – 6.94 (m, 122H), 4.97 – 4.26 (m, 186H), 1.09 (m, 2156H).

Limited solubility prevented acquisition of a ^{13}C NMR.

C. Synthesis of TIPS-Anthracene Dendrimers

To a vial was added PhBr dendrimer ([Gn]-PhBr$_x$), TIPS-anthracene-2-boronic acid pinacol ester (10 equiv. for G1/16 equiv. for G2/30 equiv. for G3/54 equiv. for G4), potassium carbonate, and Pd(dppf)Cl_2·DCM under inert atmosphere. Dry, degassed THF and degassed water were added, and the reaction was allowed to stir at 75°C for 24 h. After the mixture was cooled to room temperature, it was dried with Na_2SO_4 and concentrated under reduced pressure. Crude solid was purified by silica gel column chromatography with 0-100% DCM/hexanes eluent, followed by precipitation into methanol at 0°C to afford desired product as yellow solid.

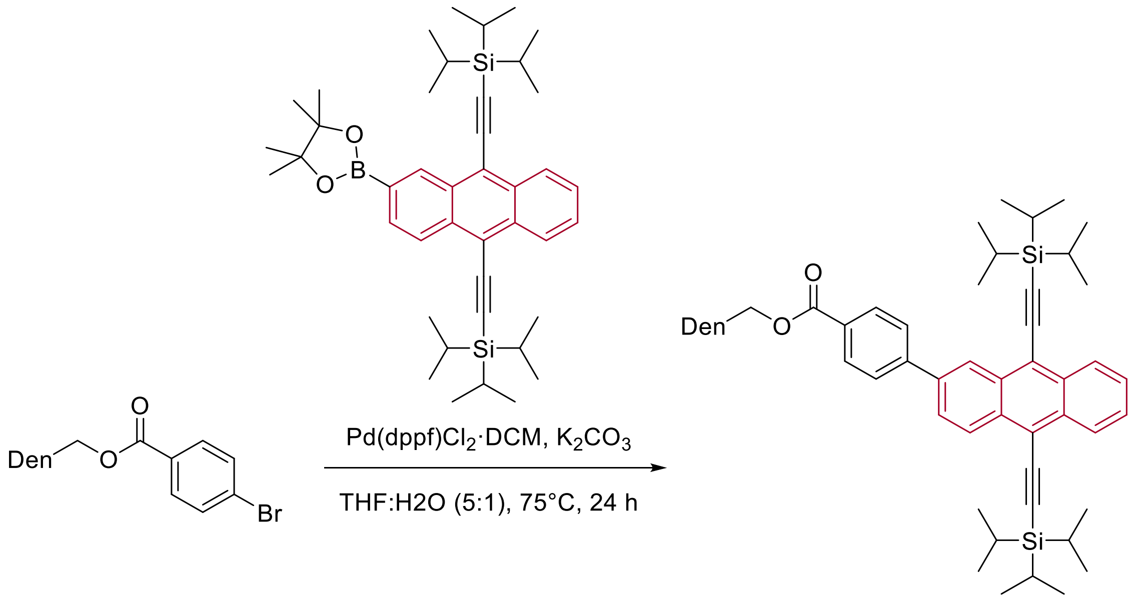

Supplementary Fig. 29. Synthesis of anthracene dendrimer.

[G1]-A_6

[G1]-$PhBr_6$ (100 mg, 0.063 mmol dendrimer, 1 equiv.), TIPS-anthracene-2-boronic acid pinacol ester (419 mg, 0.63 mmol, 10 equiv.), Pd(dppf)Cl_2·DCM (51 mg, 0.063 mmol, 1 equiv.), and potassium carbonate (174 mg, 1.26 mmol, 20 equiv.) in dry, degassed THF (5 mL) and degassed H_2O (1 mL) were used in the synthesis of [G1]-A_6 (133 mg, 49% yield).

^{1}H-NMR (400 MHz, $CDCl_3$, δ): 8.89 (s, 6H), 8.66 (d, 6H), 8.59 (dd, 12H), 8.09 (d, 12H), 7.83 (m, 18H), 7.58 (m, 12H), 4.64 (dd, 12H), 4.23 (s, 6H), 1.46 (s, 9H), 1.39 (m, 2H), 1.36-1.10 (m, 246H), 0.92 (m, 6H), 0.82 (t, 3H)

^{13}C-NMR (126 MHz, $CDCl_3$, δ): 172.22, 165.75, 145.32, 137.52, 132.75, 132.65, 132.41, 131.85, 130.23, 128.52, 128.20, 127.35, 127.29, 127.25, 127.06, 127.04, 126.13, 125.68, 119.20, 118.62, 105.49, 105.01, 103.20, 103.12, 65.72, 63.68, 47.02, 41.76, 29.72, 18.89, 18.11, 11.49, 11.46, 7.25

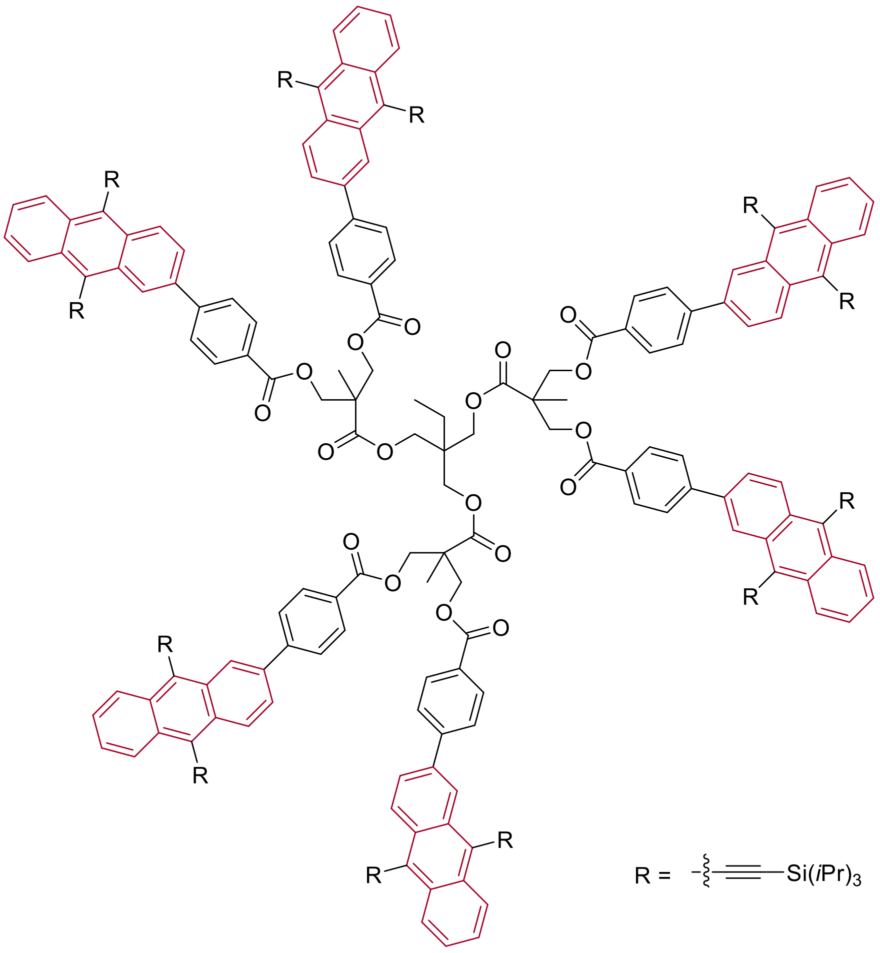

[G2]-A_{12}

[G2]-$PhBr_{12}$ (100 mg, 0.03 mmol dendrimer, 1 equiv.), TIPS-anthracene-2-boronic acid pinacol ester (319 mg, 0.48 mmol, 16 equiv.), Pd(dppf)Cl_2·DCM (49 mg, 0.06 mmol, 2 equiv.), and potassium carbonate (133 mg, 0.96 mmol, 32 equiv.) in dry, degassed THF (5 mL) and degassed H_2O (1 mL) were used in the synthesis of [G2]-A_{12} (127 mg, 48% yield).

^{1}H-NMR (400 MHz, $CDCl_3$, δ): 8.86 (s, 12H), 8.71-8.54 (m, 36H), 8.10 (d, 24H), 7.82 (d, 36H), 7.57 (m, 24H), 4.77-4.42 (m, 36H), 4.24 (s, 6H), 1.47 (s, 18H), 1.42-1.37 (m, 11H), 1.35-1.09 (m, 504H), 0.91 (m, 3H)

^{13}C-NMR (126 MHz, $CDCl_3$, δ): 172.06, 171.73, 165.69, 145.16, 137.52, 132.70, 132.60, 132.38, 131.80, 130.22, 128.60, 128.15, 127.28, 127.25, 127.00, 126.97, 126.11, 125.61, 119.16, 118.57, 105.43, 104.92, 103.18, 103.11, 65.58, 65.37, 64.25, 46.97, 46.87, 41.58, 29.74, 18.85, 18.09, 17.72, 11.46, 11.41, 7.61

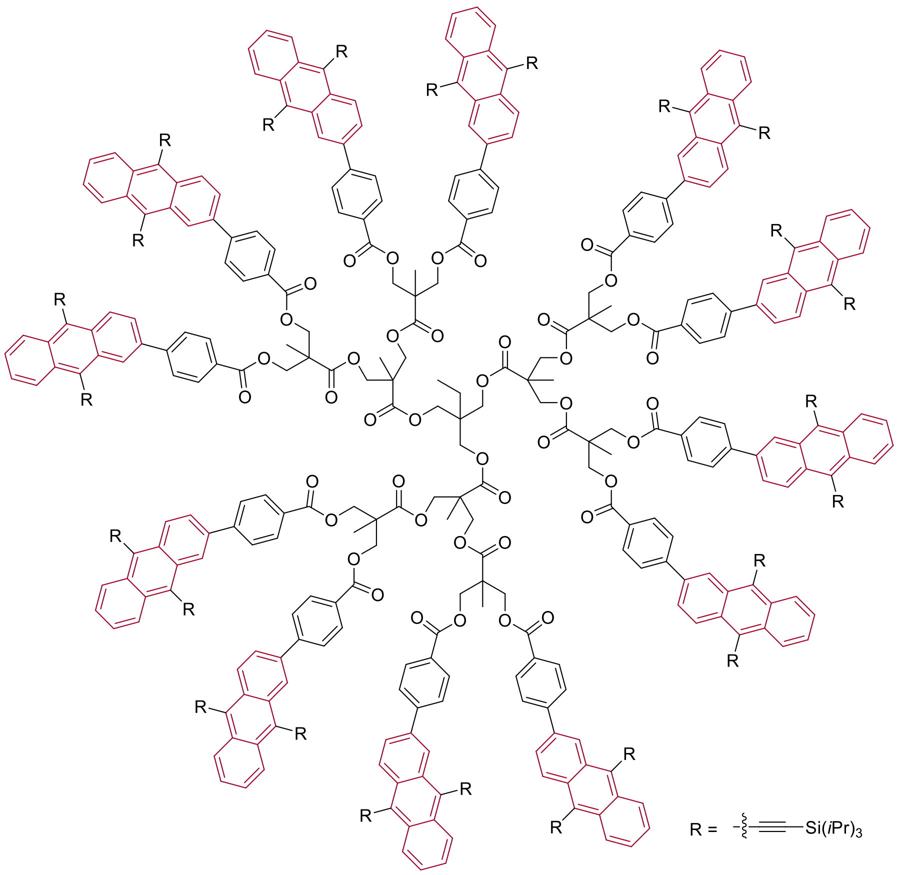

[G3]-A_{24}

[G3]-$PhBr_{24}$ (100 mg, 0.014 mmol dendrimer, 1 equiv.), TIPS-anthracene-2-boronic acid pinacol ester (279 mg, 0.42 mmol, 30 equiv.), Pd(dppf)Cl_2·DCM (46 mg, 0.056 mmol, 4 equiv.), and potassium carbonate (116 mg, 0.84 mmol, 60 equiv.) in dry, degassed THF (5 mL) and degassed H_2O (1 mL) were used in the synthesis of [G3]-A_{24} (171 mg, 68% yield).

^{1}H-NMR (400 MHz, $CDCl_3$, δ): 8.77 (m, 24H), 8,52 (m, 72H), 8.05 (m, 48H), 7.74 (m, 72H), 7.52 (m, 48H), 4.87-4.26 (m, 90H), 1.32-1.03 (m, 1076H)

Due to poor solubility, ^{13}C-NMR was not collected.

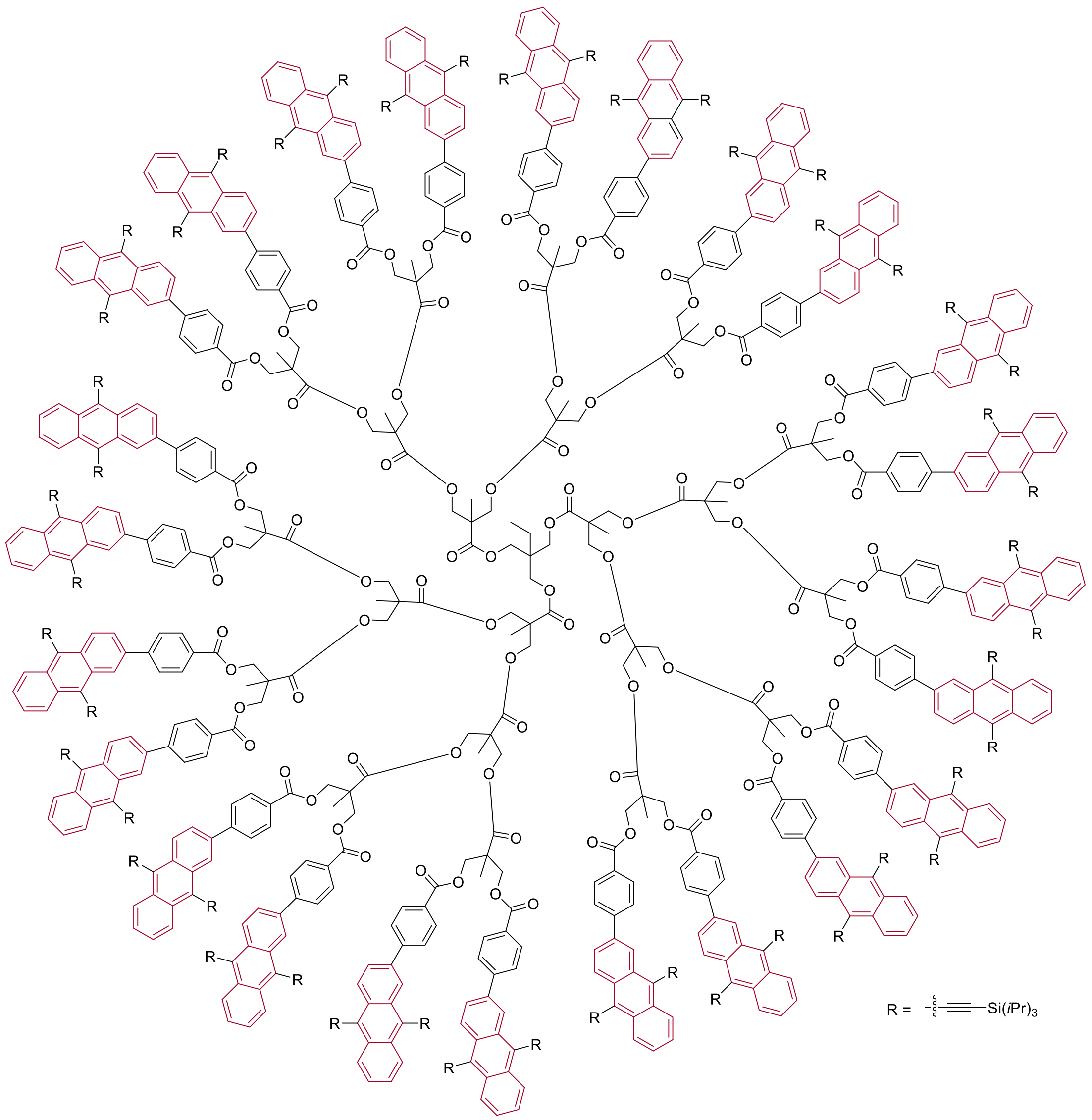

[G4]-A_{48}

[G4]-$PhBr_{48}$ (100 mg, 0.0071 mmol dendrimer, 1 equiv.), TIPS-anthracene-2-boronic acid pinacol ester (255 mg, 0.38 mmol, 54 equiv.), Pd(dppf)Cl_2·DCM (47 mg, 0.057 mmol, 8 equiv.), and potassium carbonate (106 mg, 0.77 mmol, 108 equiv.) in dry, degassed THF (5 mL) and degassed H_2O (1 mL) were used in the synthesis of [G4]-A_{48} (150 mg, 58% yield).

^{1}H-NMR (400 MHz, $CDCl_3$, δ): 8.64 (m, 48H), 8.43 (m, 144H), 7.97 (m, 98H), 7.63 (m, 144H), 7.43 (m, 98H)

Due to poor solubility, ^{13}C-NMR was not collected.

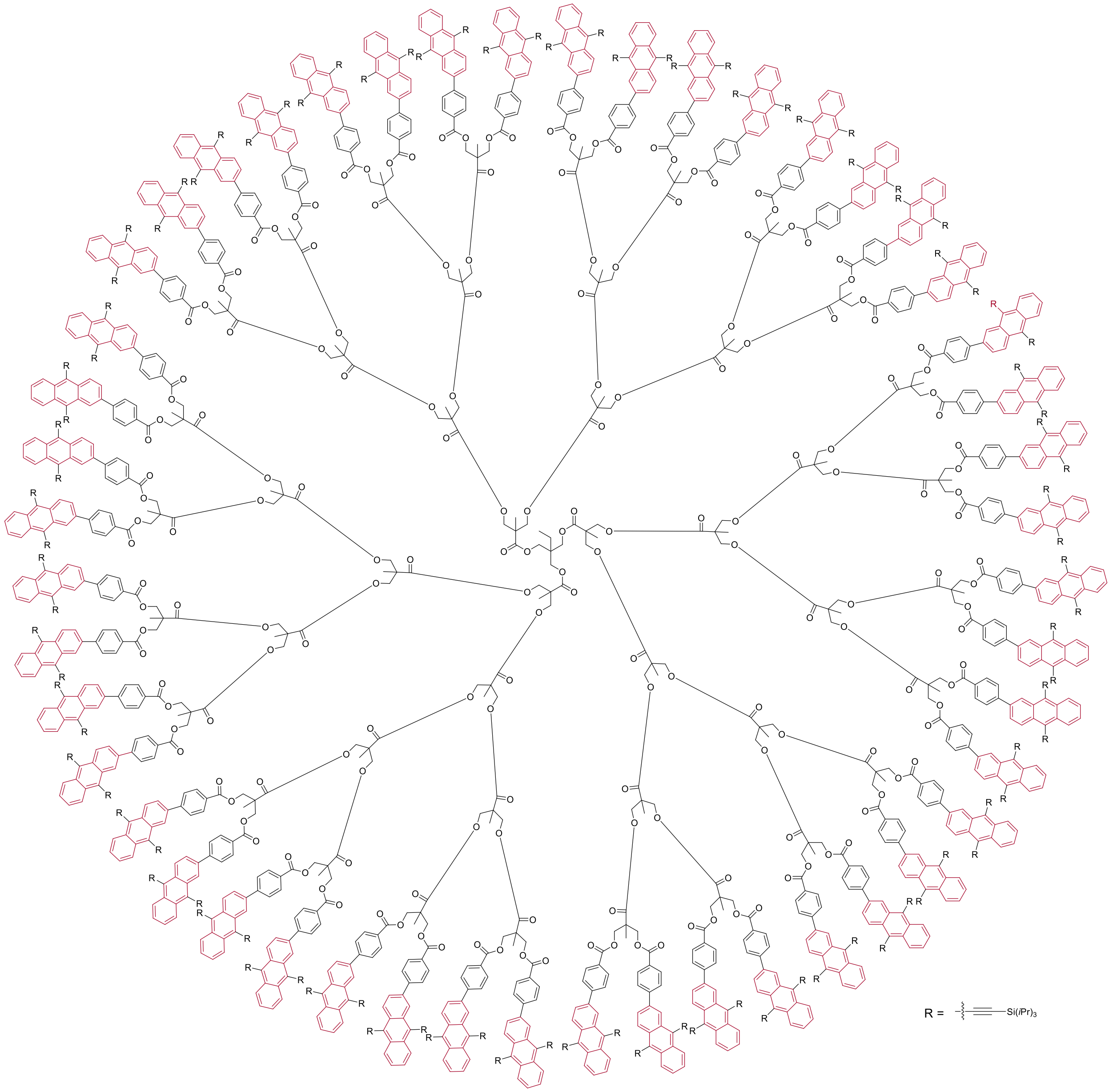

III. Supplementary Notes

A. MALDI Spectra

Supplementary Table 3. MALDI details

Dendrimer	Matrix	Ion	Calculated Mass	Observed Mass	Summary
[G1]-Ph_6	DCTB	-	1573.9	1605.3 (+Na)<br>1621.2 (+K)	complete growth
[G2]-Ph_{12}	DHB	NaTFA	3361.8	3386.2	complete growth
[G3]-Ph_{24}	DHB	-	6937.6	6963.1	complete growth
[G4]-Ph_{48}	DHB	NaTFA	14089.2	14163 (+Na)	complete growth
[G1]-P_6	DCTB	NaTFA	4924.6	4930	complete growth
[G2]-P_{12}	DCTB	-	10063.2	10070.7<br>10092.3 (+Na)<br>9504.3 (incomplete growth)	99% complete
[G3]-P_{24}	DCTB	NaTFA*	20340.4	20640.2<br>19980.1/20065.6<br>19425.4/19608.4	possibly 2 groups incomplete
[G4]-P_{48}	DCTB	NaTFA*	40894.8	33000-41000	90% complete based on average highest peak
[G1]-A_6	DHB	NaTFA*	4324.37	4350.74 (M+Na)<br>4366.73 (M+K)	complete functionalization
[G2]-A_{12}	DCTB	NaTFA*	8862.79	8866.78 (M+H)<br>8889.99 (M+Na)	complete functionalization
[G3]-A_{24}	DCTB	NaTFA	17939.62	17975.17 (M+K)<br>17334.14/16694.43	up to 2 groups hydrolyzed
[G4]-A_{48}	DCTB	NaTFA	36091.32	36134.52 (M+K)<br>35702.29<br>35053.66<br>34616.81	up to 3 groups hydrolyzed

Samples were premixed in an Eppendorf in a 40:1:1 Matrix: Sample: Ion ratio (in mass). THF is the solvent for all entries.

*40:2:1 Matrix: Sample: Ion ratio (in mass)

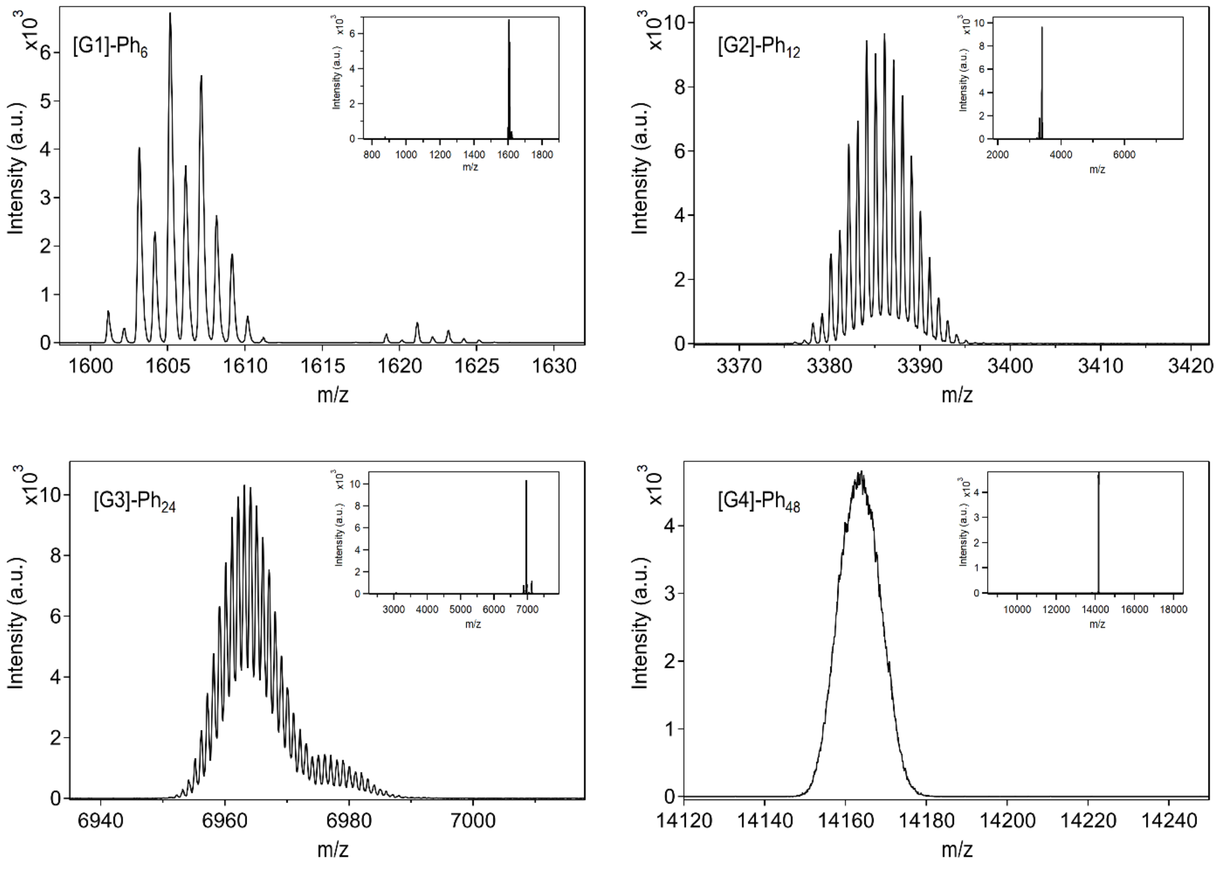

Supplementary Fig. 30. MALDI spectra of Phenyl dendrimers.

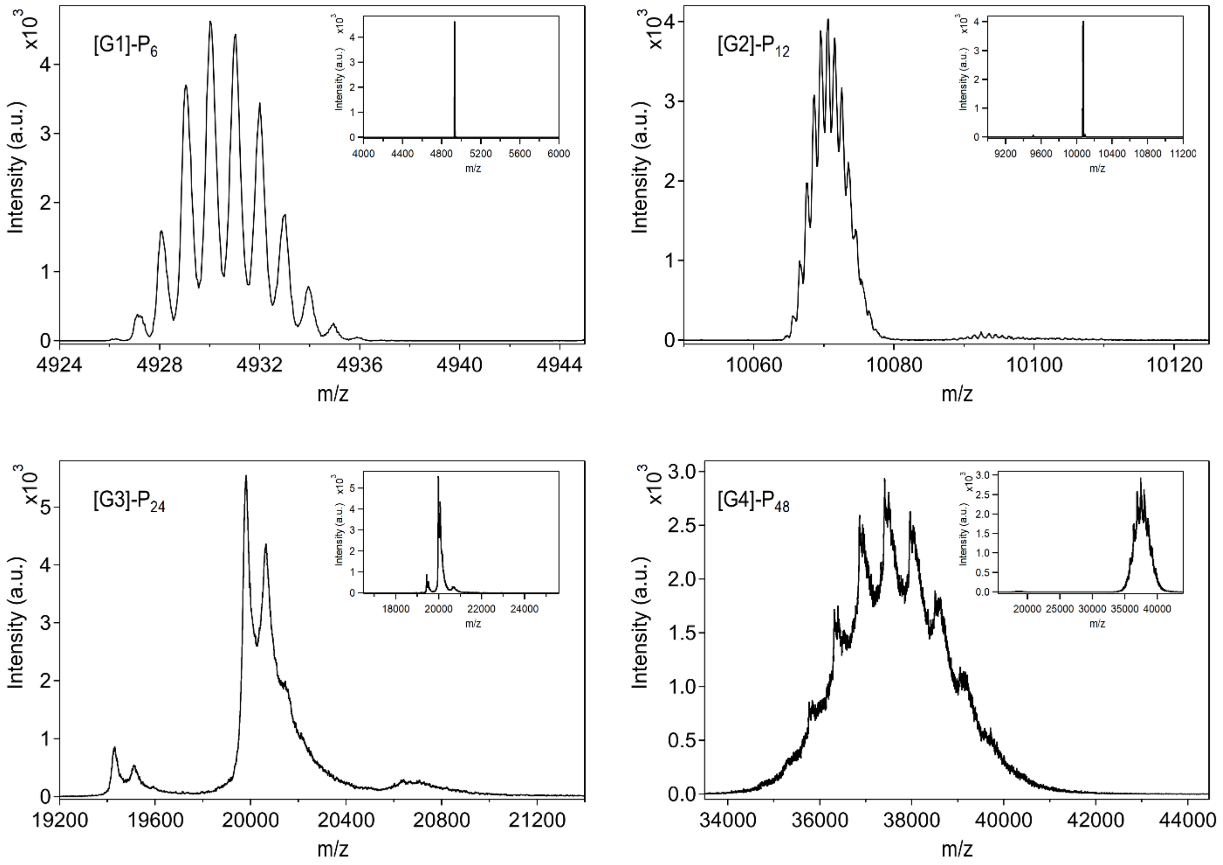

Supplementary Fig. 31. MALDI spectra of Pentacene dendrimers.

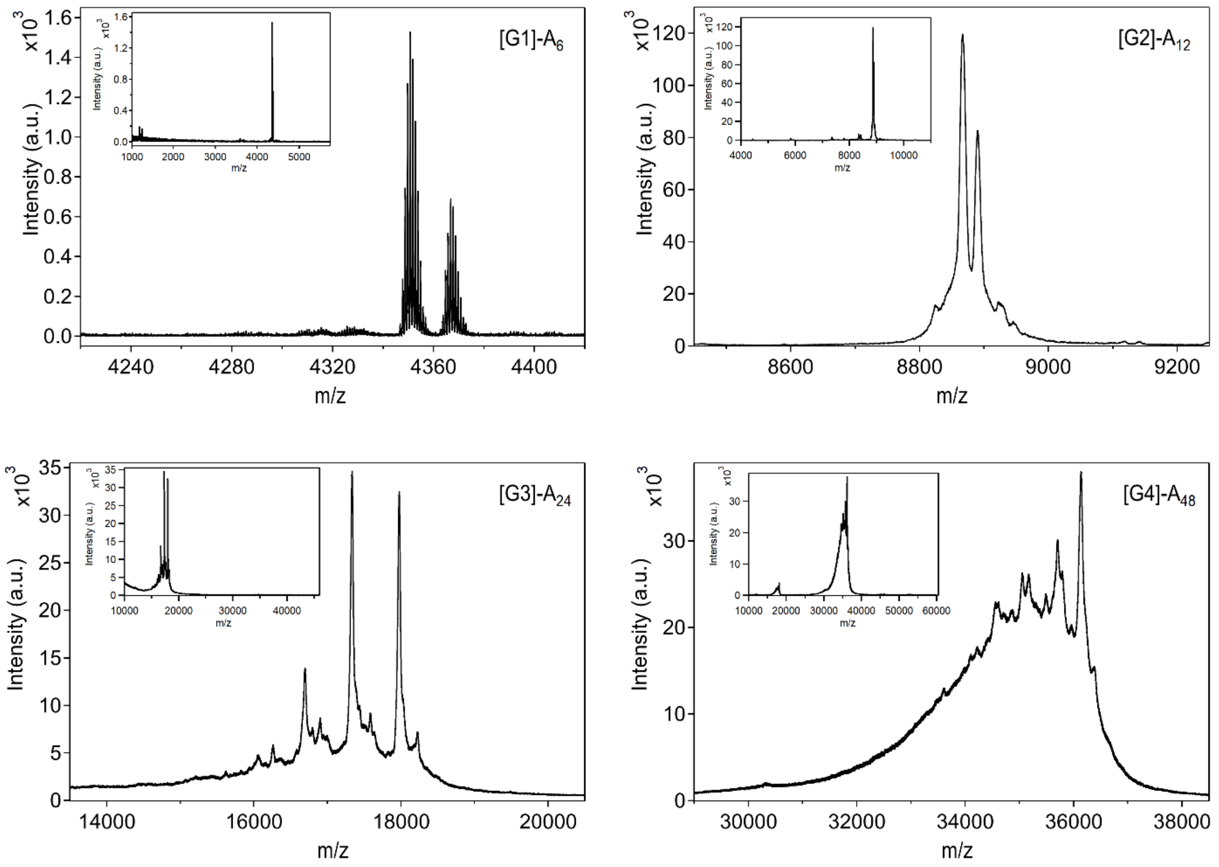

Supplementary Fig. 32. MALDI spectra of Anthracene dendrimers.

B. GPC Data

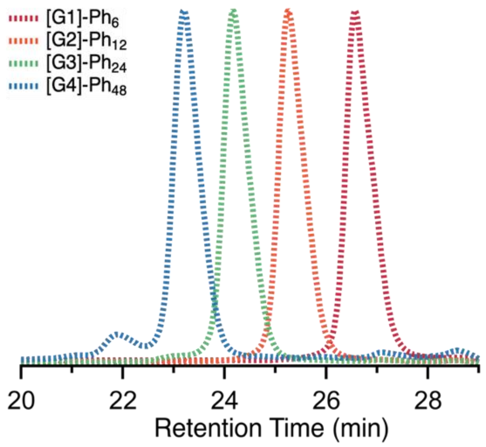

Supplementary Fig. 33. GPC traces of PhBr dendrimers.

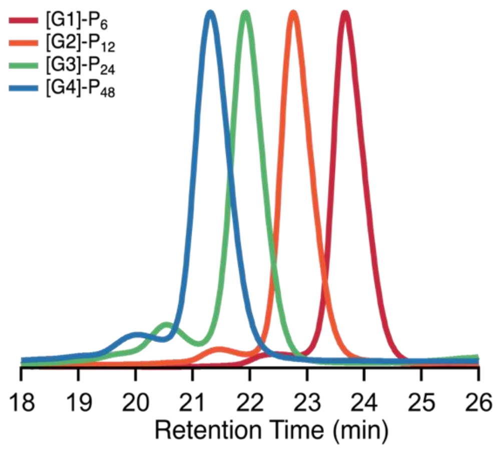

Supplementary Fig. 34. GPC traces of Pentacene dendrimers.

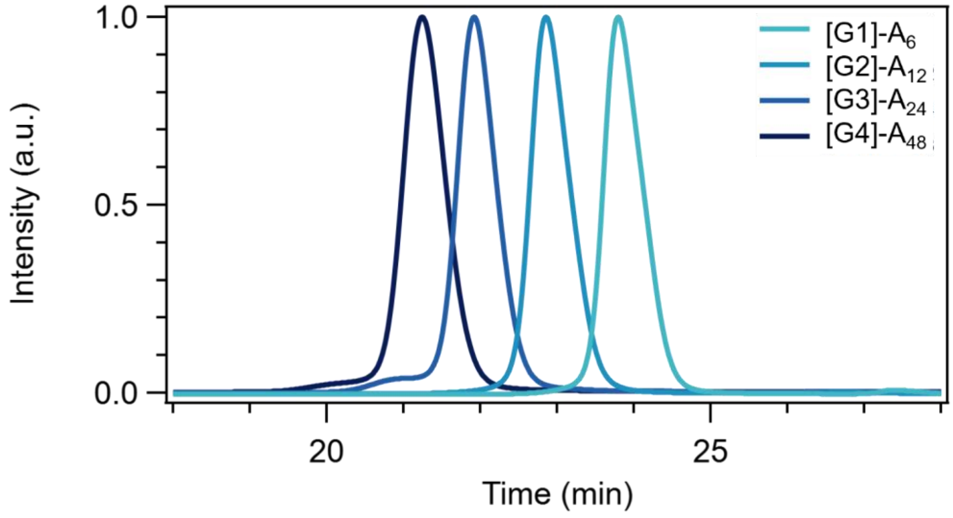

Supplementary Fig. 35. GPC traces of Anthracene dendrimers.

Supplementary Table 4. Dispersity data obtained from GPC.

**Dendrimer**	***Đ***
[G1]-Ph_6	1.03
[G2]-Ph_{12}	1.02
[G3]-Ph_{24}	1.01
[G4]-Ph_{48}	1.03
[G1]-P_6	1.02
[G2]-P_{12}	1.02
[G3]-P_{24}	1.05
[G4]-P_{48}	1.04
[G1]-A_6	1.01
[G2]-A_{12}	1.01
[G3]-A_{24}	1.02
[G4]-A_{48}	1.02

C. NMR Spectra

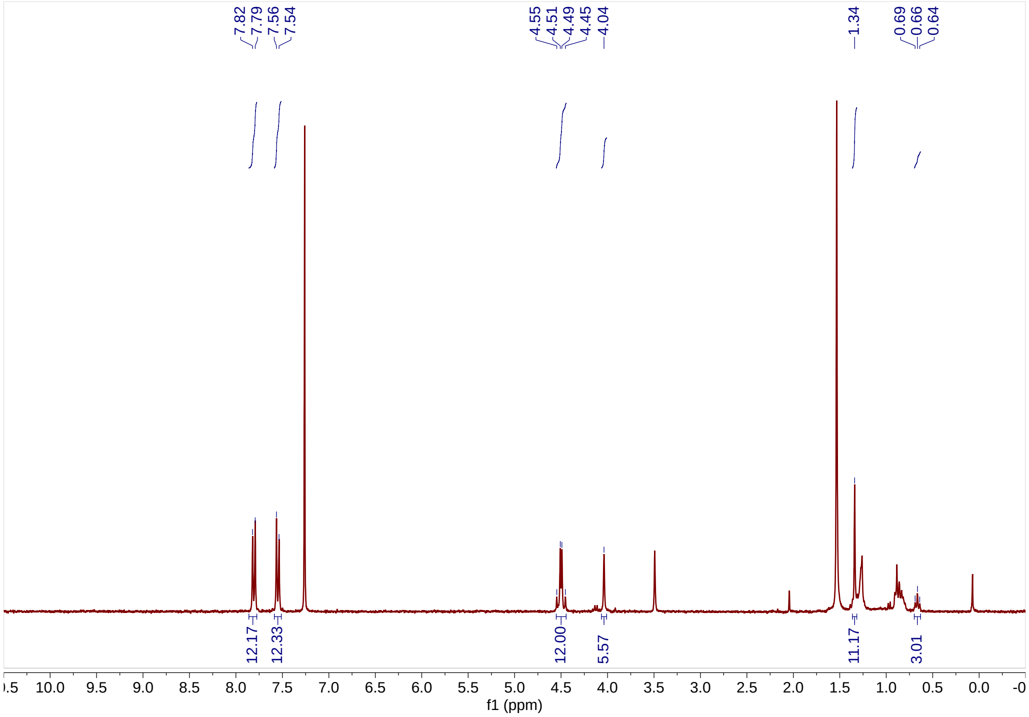

Supplementary Fig. 36. ^{1}H-NMR spectrum (300 MHz) of [G1]-Ph_6 in $CDCl_3$.

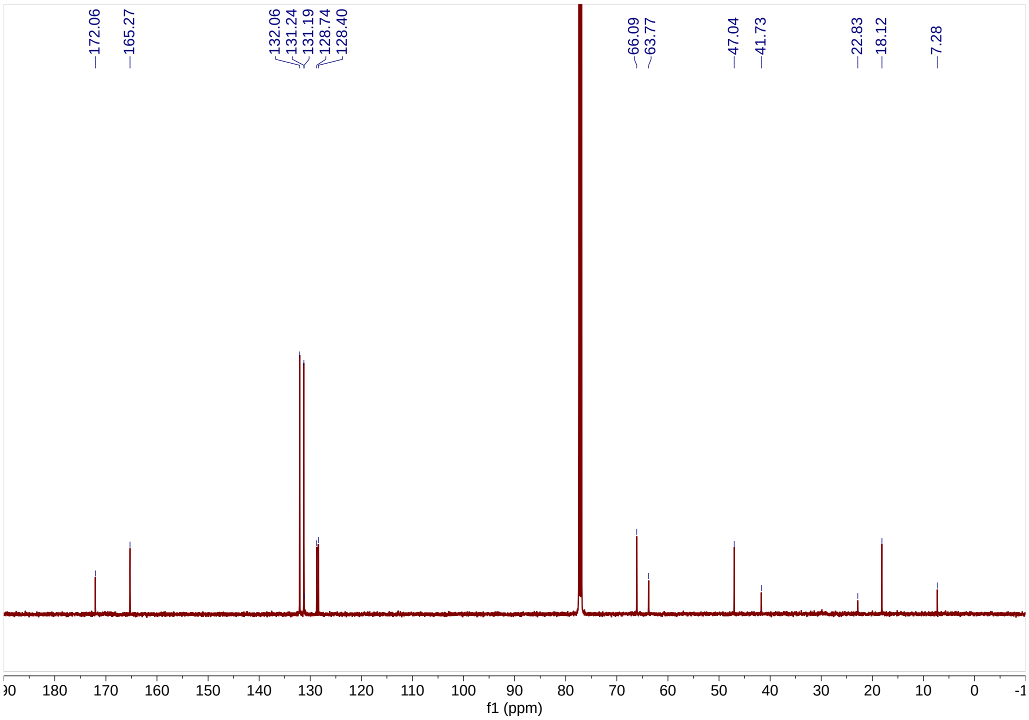

Supplementary Fig. 37. ^{13}C-NMR spectrum (126 MHz) of [G1]-Ph_6 in $CDCl_3$.

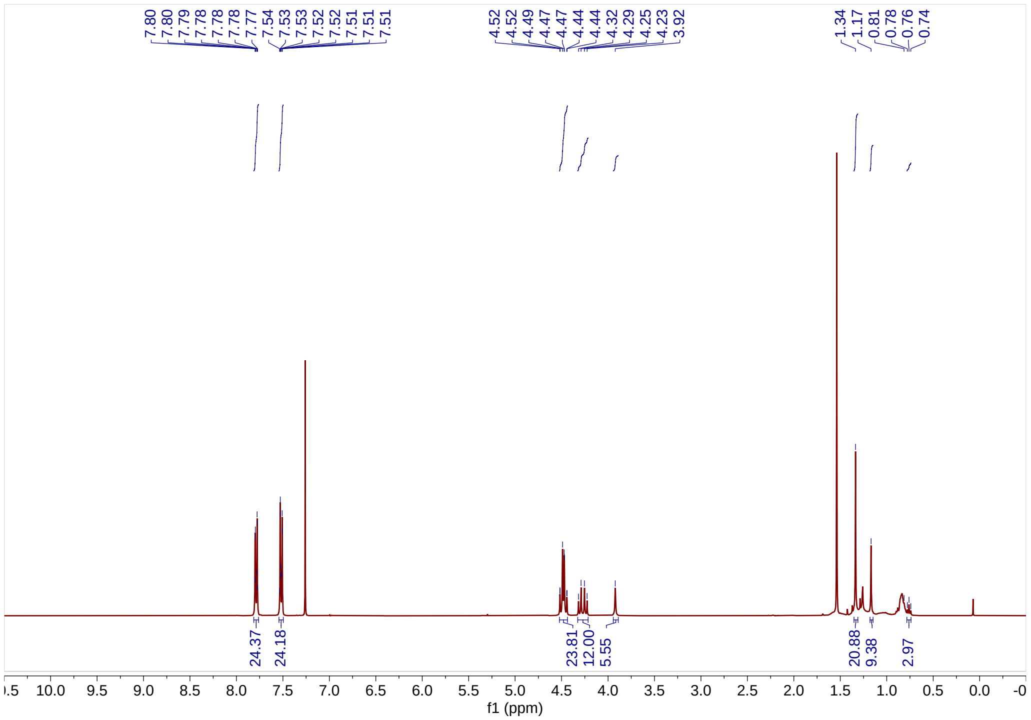

Supplementary Fig. 38. ^{1}H-NMR spectrum (400 MHz) of [G2]-Ph_{12} in $CDCl_3$.

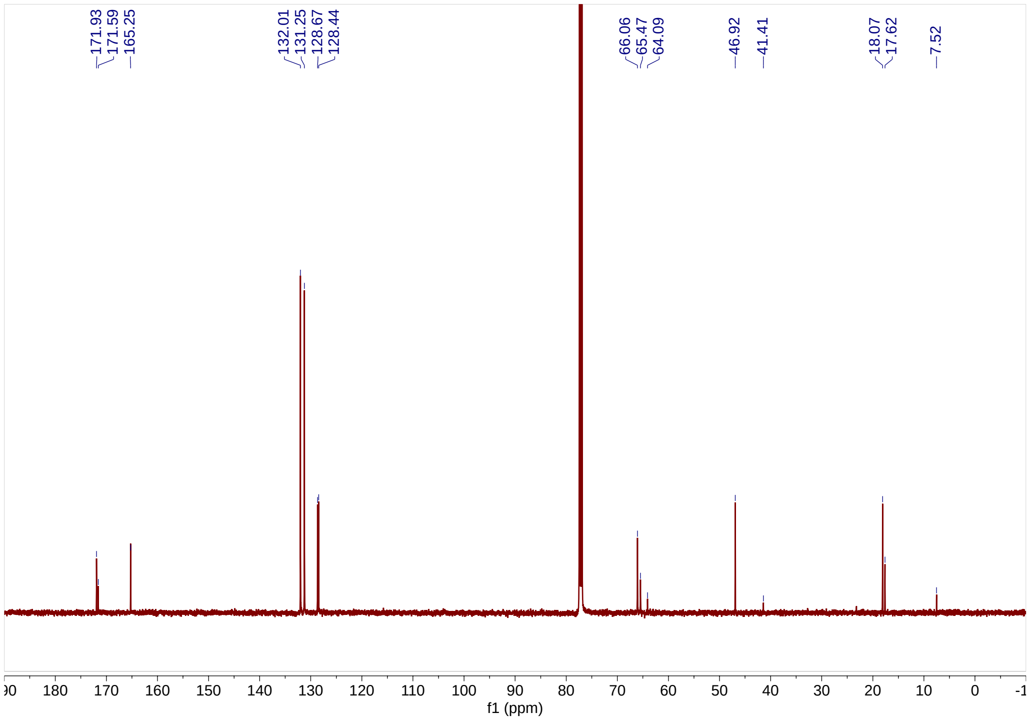

Supplementary Fig. 39. ^{13}C-NMR spectrum (126 MHz) of [G2]-Ph_{12} in $CDCl_3$.

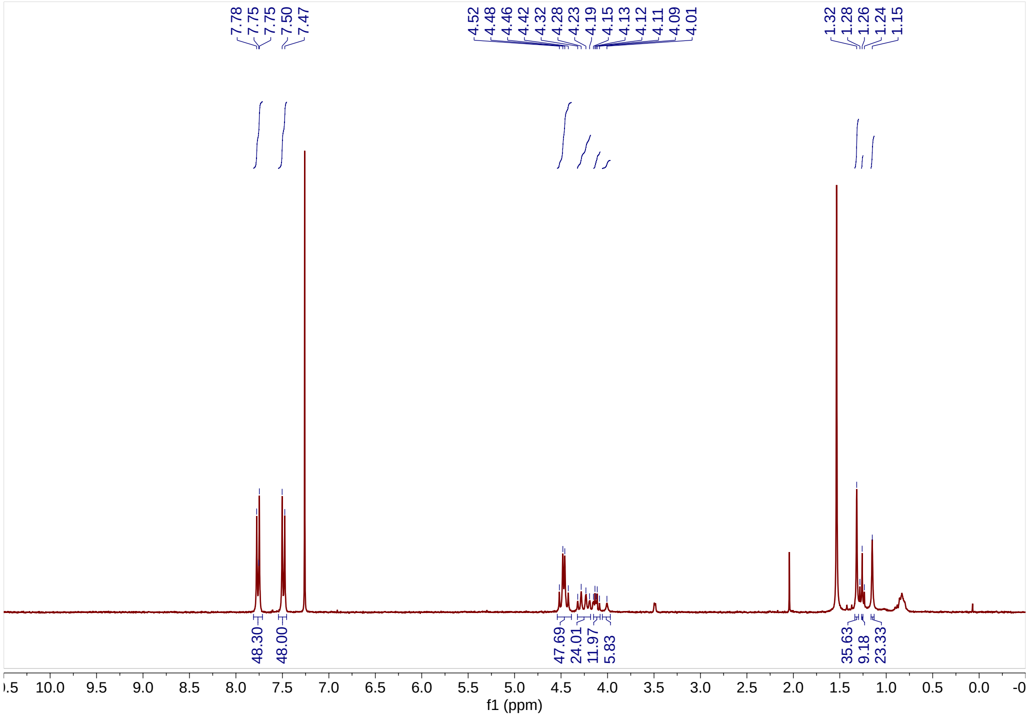

Supplementary Fig. 40. ^{1}H-NMR spectrum (300 MHz) of [G3]-Ph_{24} in $CDCl_3$.

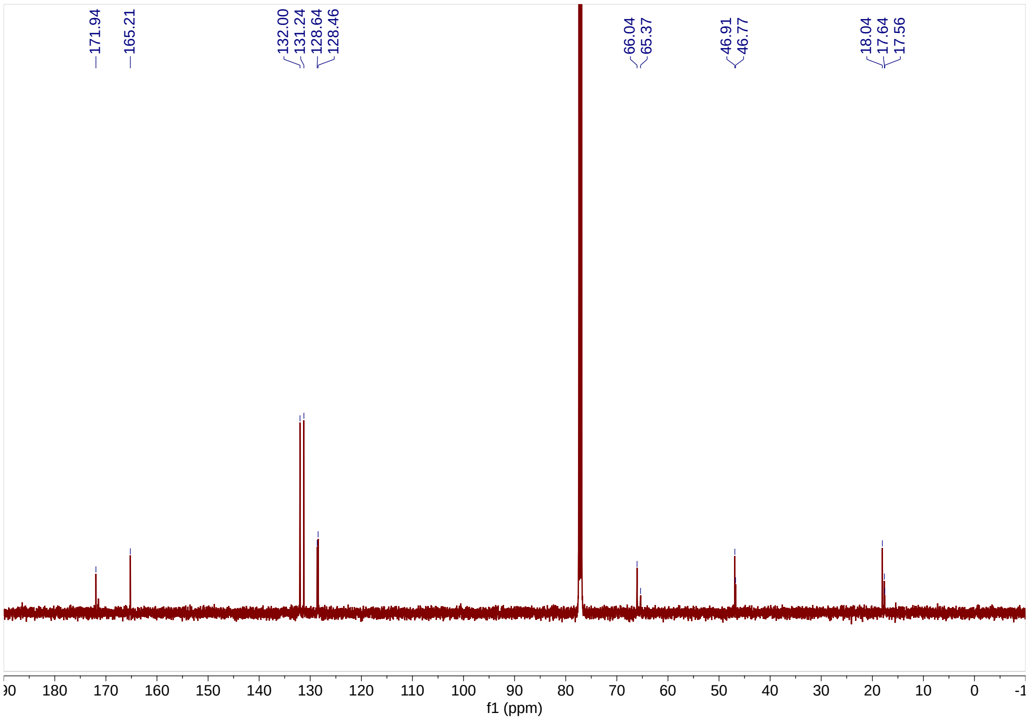

Supplementary Fig. 41. ^{13}C-NMR spectrum (126 MHz) of [G3]-Ph_{24} in $CDCl_3$.

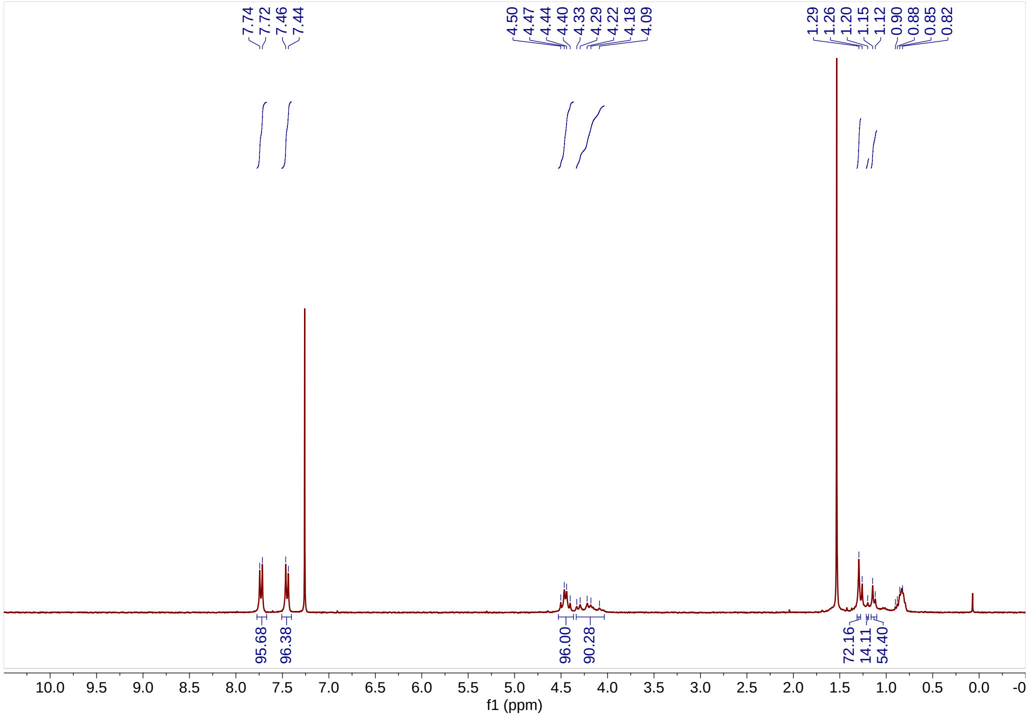

Supplementary Fig. 42. ^{1}H-NMR spectrum (300 MHz) of [G4]-Ph_{48} in $CDCl_3$.

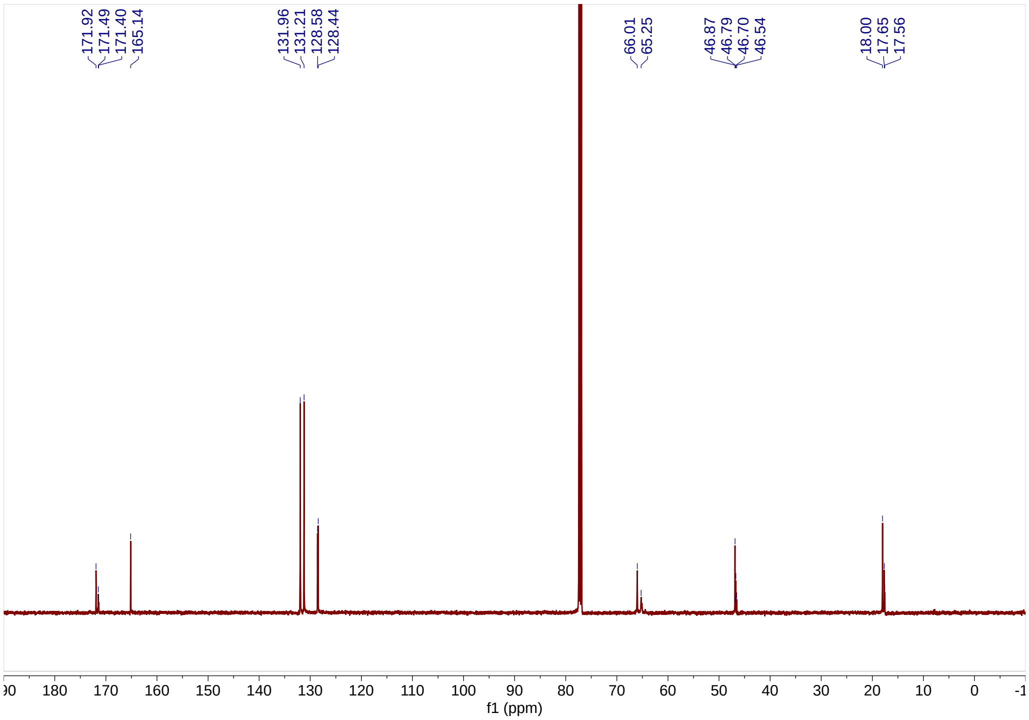

Supplementary Fig. 43. ^{13}C-NMR spectrum (126 MHz) of [G4]-Ph_{48} in $CDCl_3$.

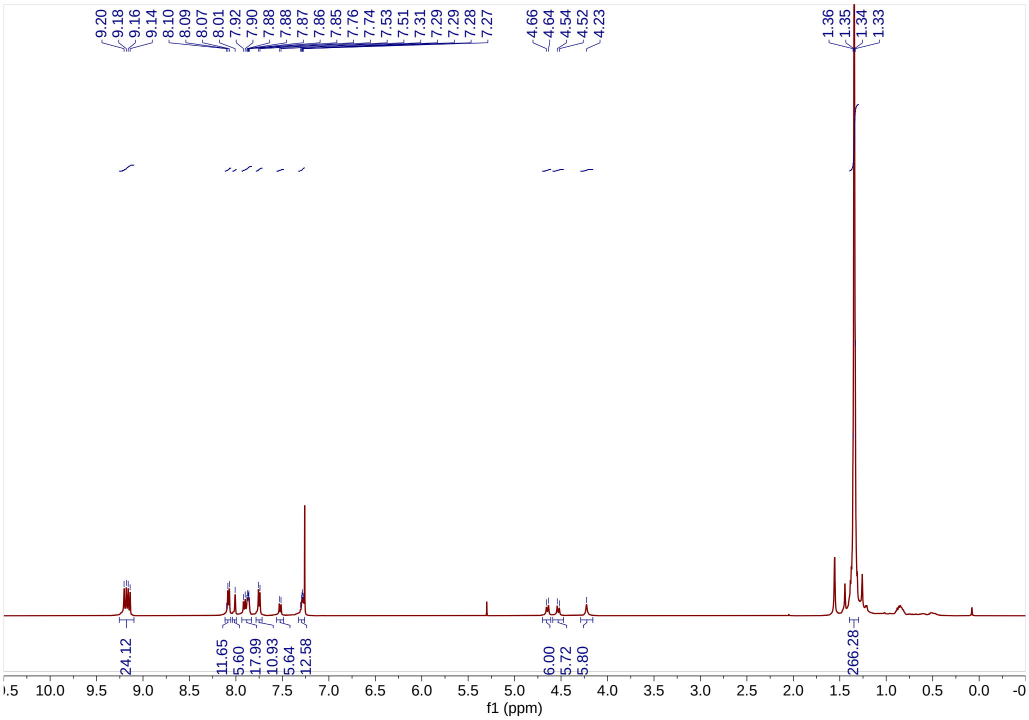

Supplementary Fig. 44. ^{1}H-NMR spectrum (500 MHz) of [G1]-P_6 in $CDCl_3$.

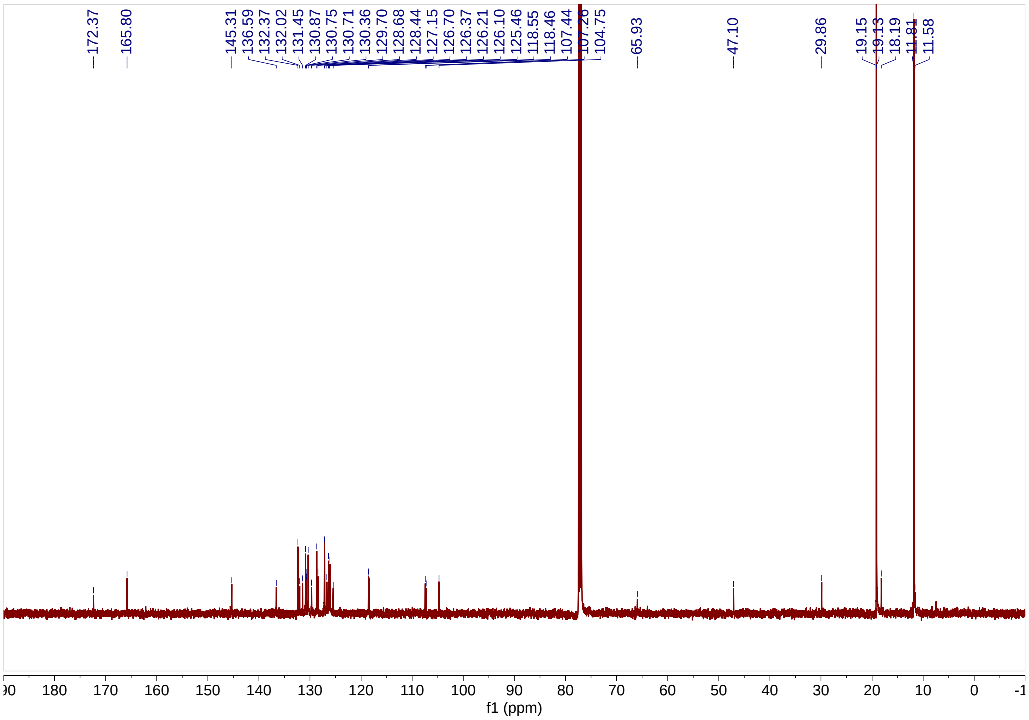

Supplementary Fig. 45. ^{13}C-NMR spectrum (126 MHz) of [G1]-P_6 in $CDCl_3$.

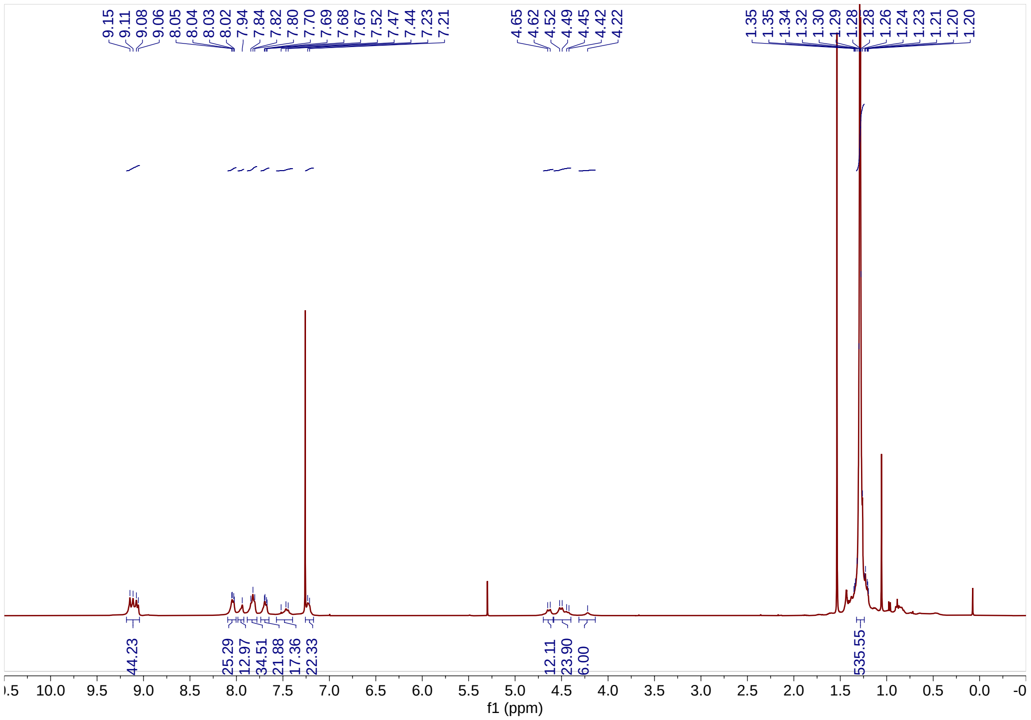

Supplementary Fig. 46. ^{1}H-NMR spectrum (400 MHz) of [G2]-P_{12} in $CDCl_3$.

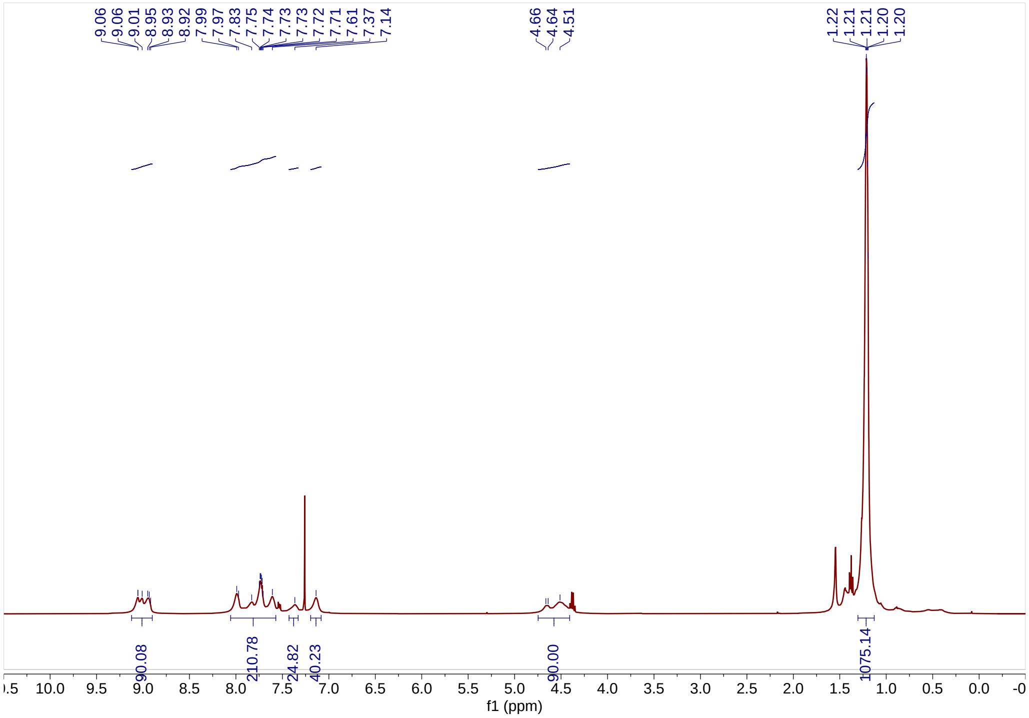

Supplementary Fig. 47. ^{1}H-NMR spectrum (400 MHz) of [G3]-P_{24} in $CDCl_3$.

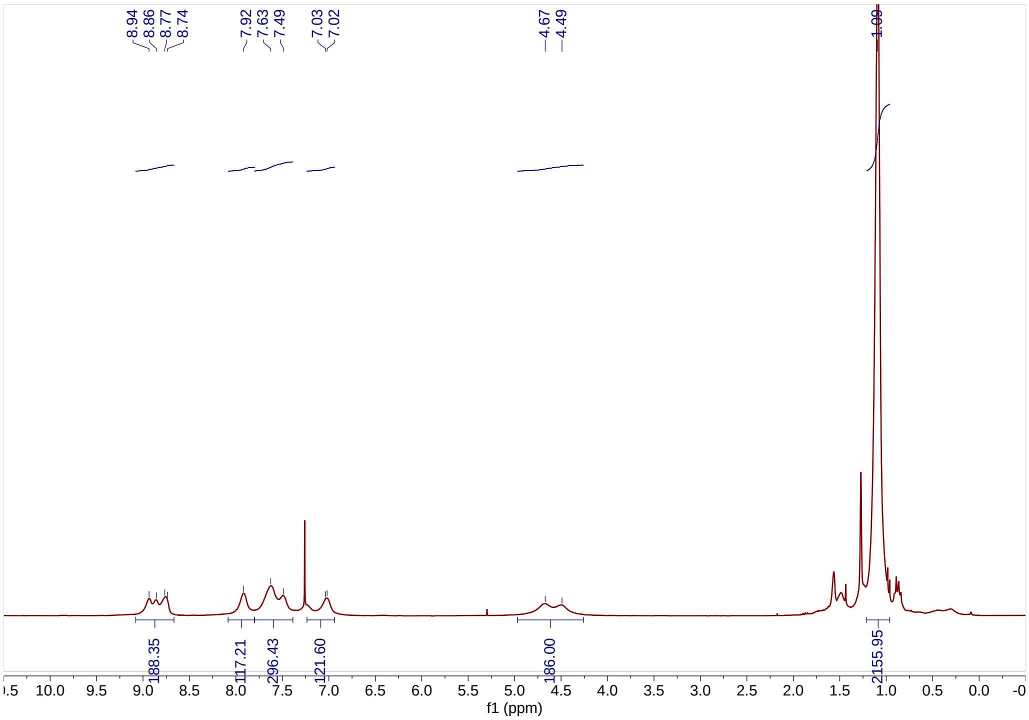

Supplementary Fig. 48. ^{1}H-NMR spectrum (300 MHz) of [G4]-P_{48} in $CDCl_3$.

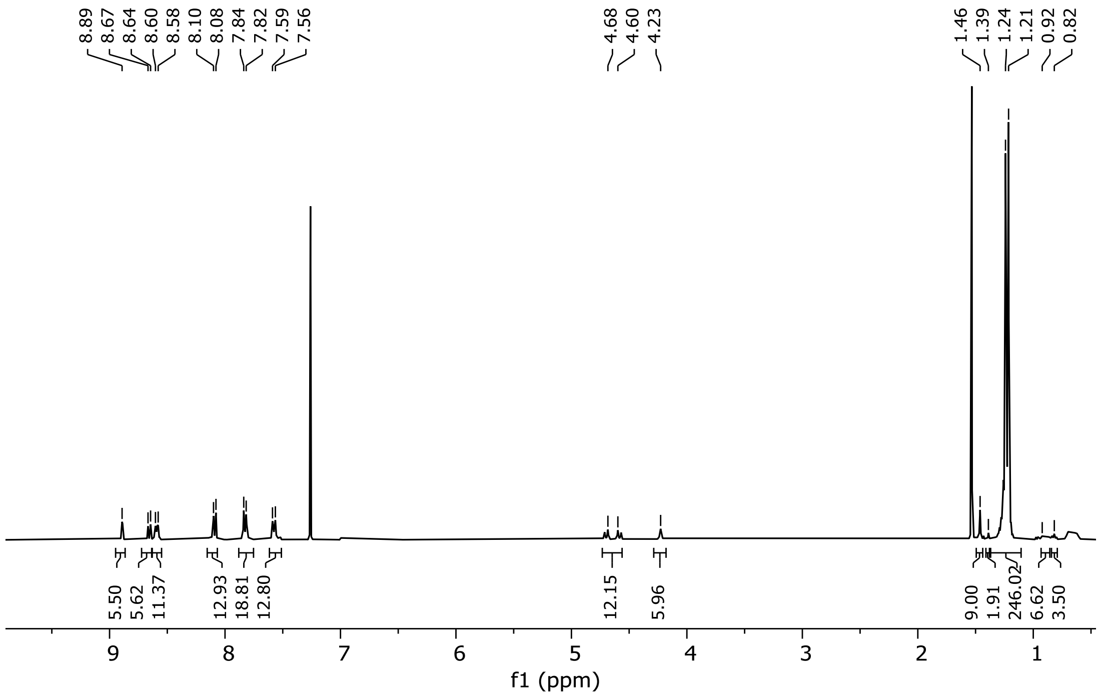

Supplementary Fig. 49. ^{1}H-NMR spectrum (400 MHz) of [G1]-A_6 in $CDCl_3$.

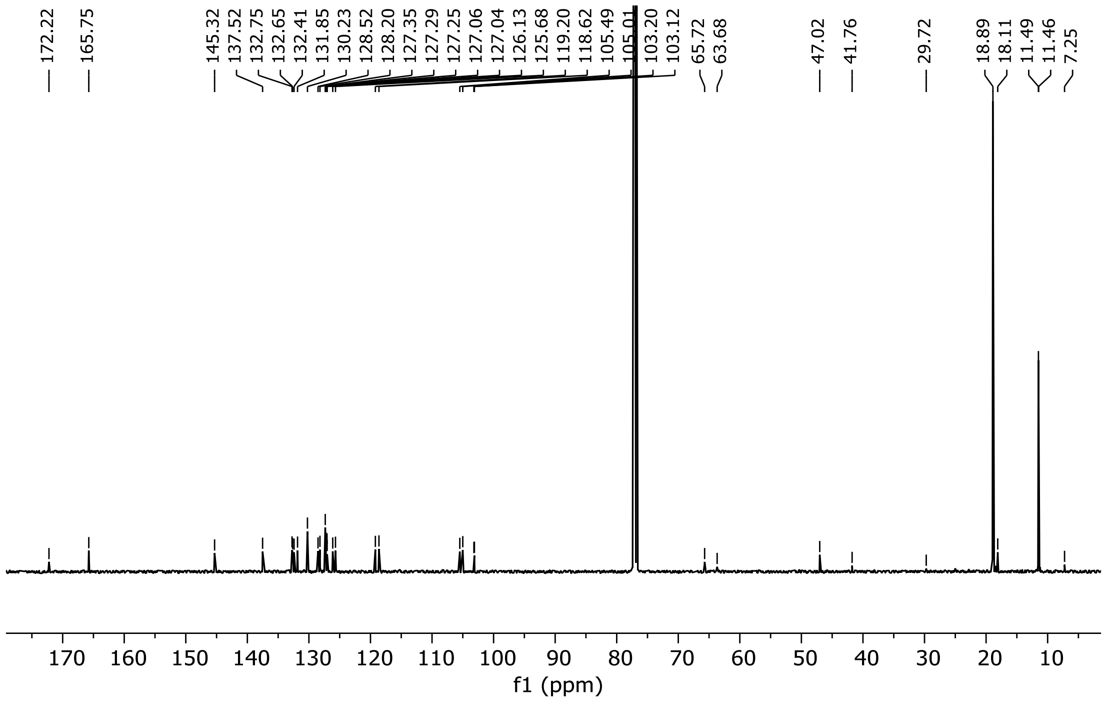

Supplementary Fig. 50. ^{13}C-NMR spectrum (126 MHz) of [G1]-A_6 in $CDCl_3$.

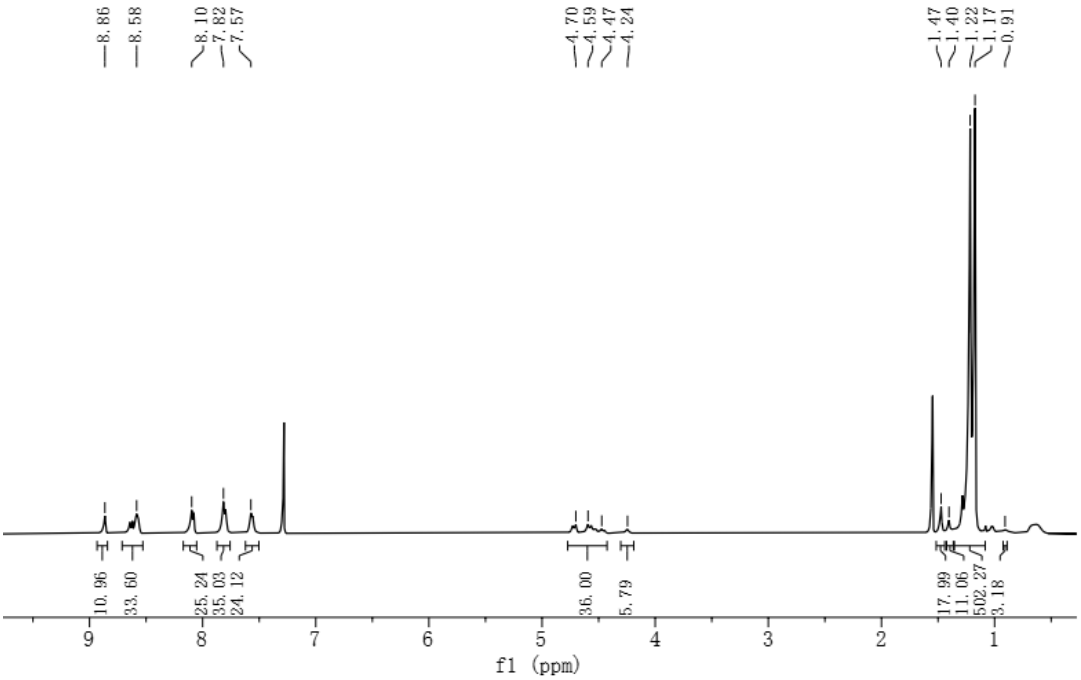

Supplementary Fig. 51. ^{1}H-NMR spectrum (400 MHz) of [G2]-A_{12} in $CDCl_3$.

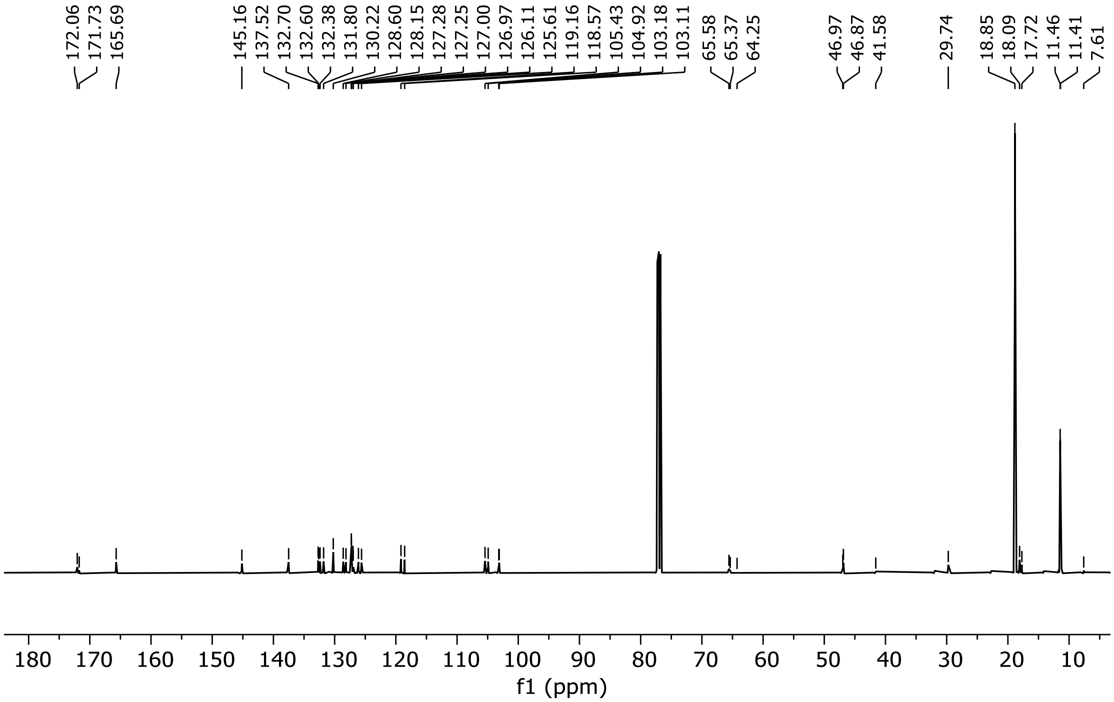

Supplementary Fig. 52. ^{13}C-NMR spectrum (126 MHz) of [G1]-A_{12} in $CDCl_3$.

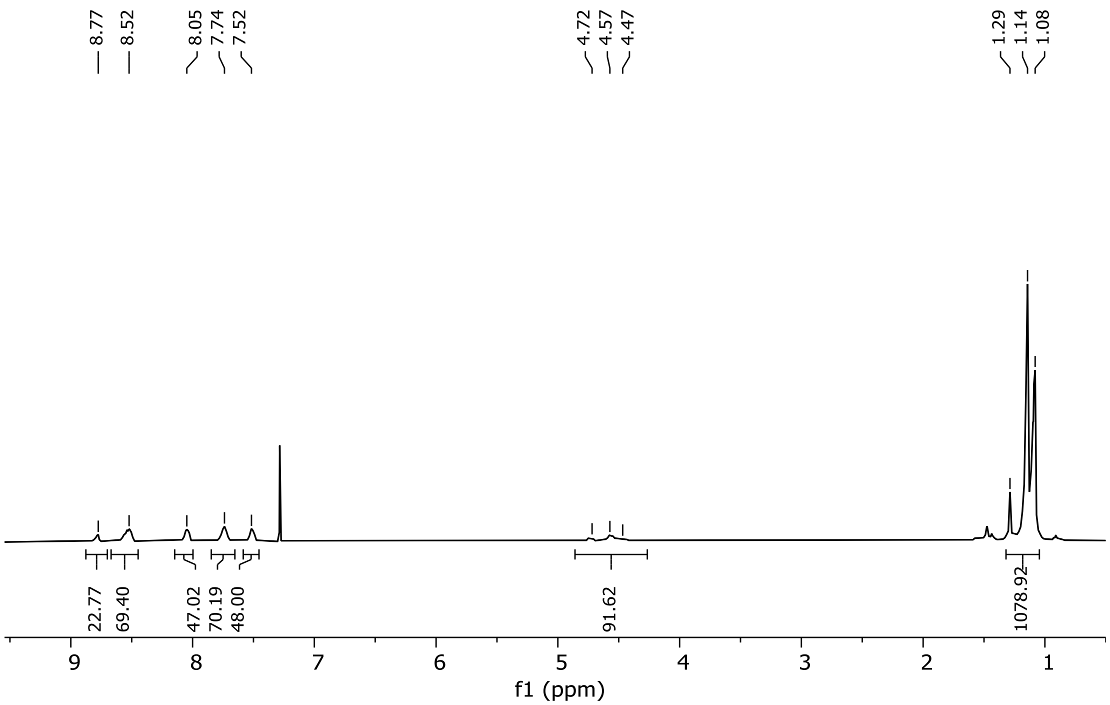

Supplementary Fig. 53. ^{1}H-NMR spectrum (400 MHz) of [G3]-A_{24} in $CDCl_3$.

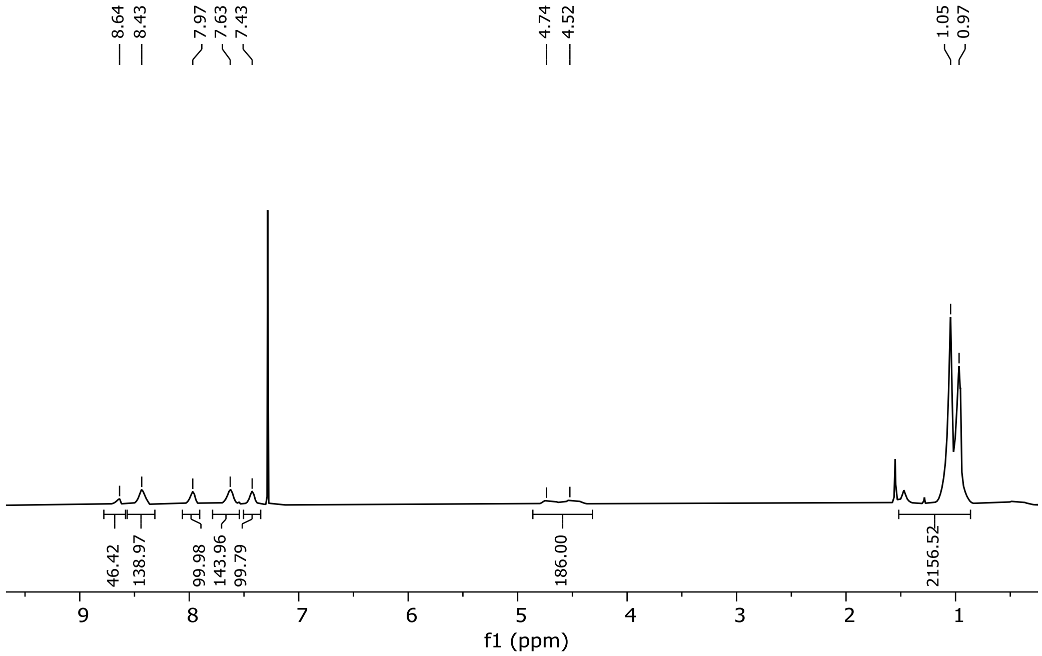

Supplementary Fig. 54. ^{1}H-NMR spectrum (400 MHz) of [G4]-A_{48} in $CDCl_3$.

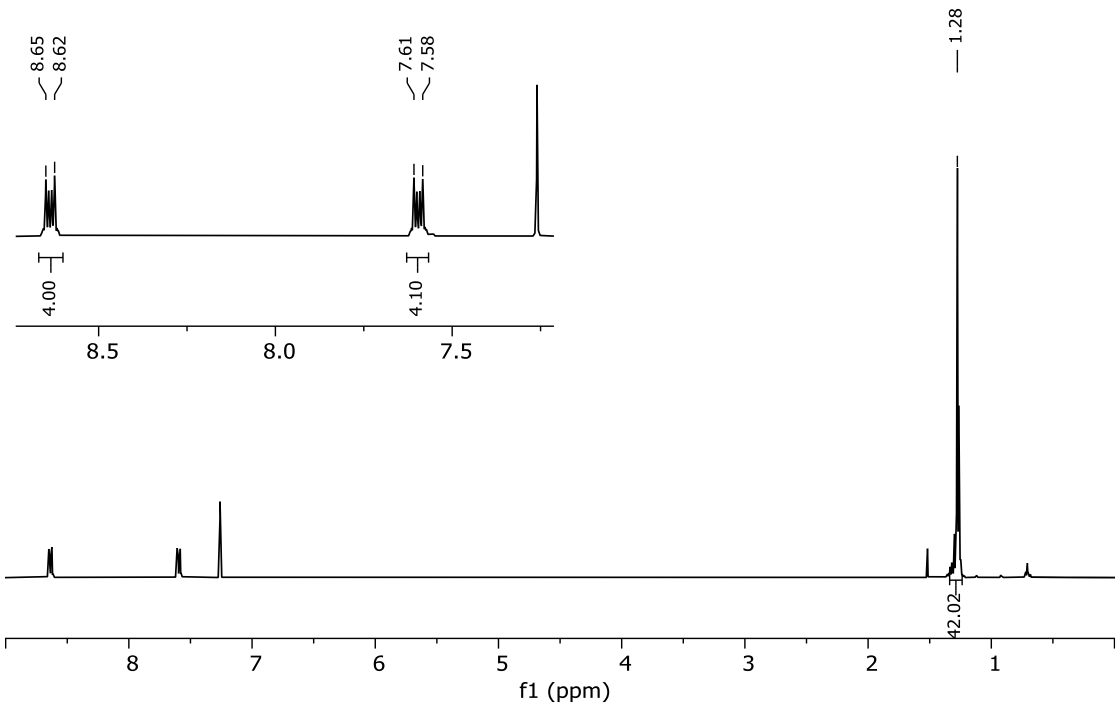

Supplementary Fig. 55. ^{1}H-NMR spectrum (400 MHz) of TIPS-An in $CDCl_3$.

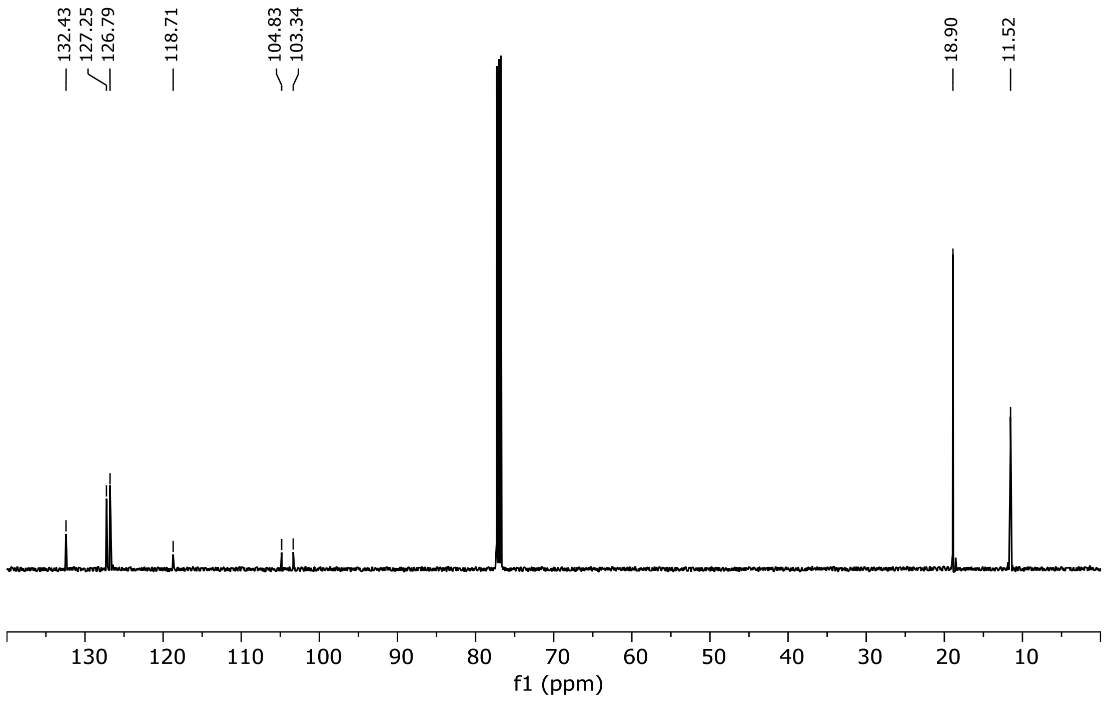

Supplementary Fig. 56. ^{13}C-NMR spectrum (126 MHz) of TIPS-An in $CDCl_3$.

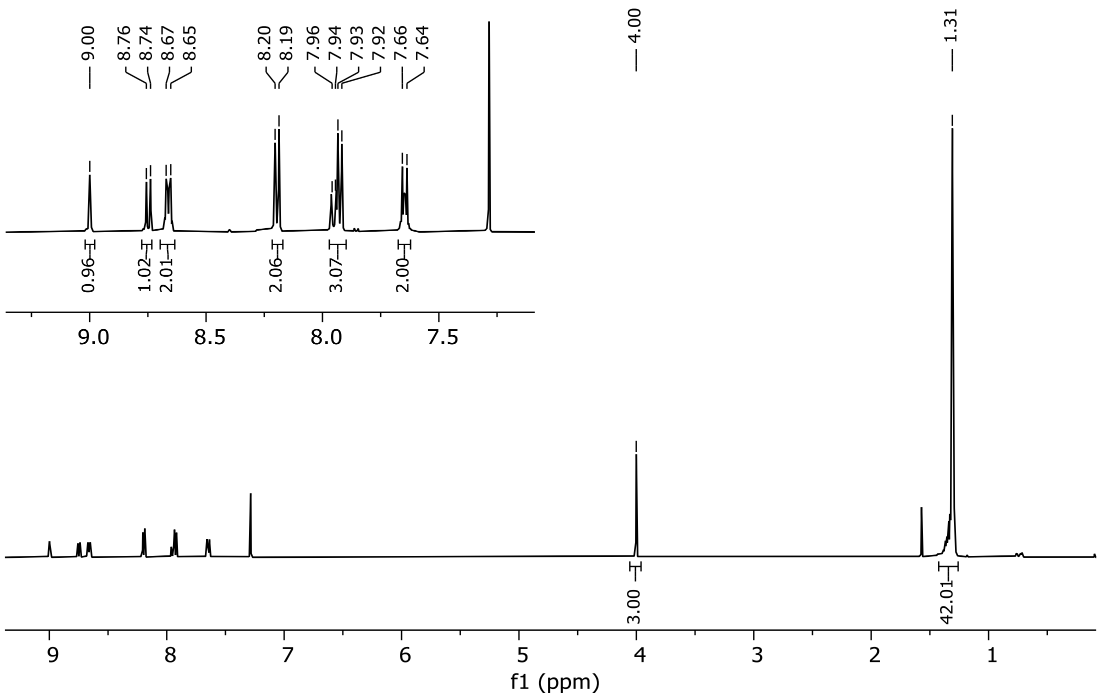

Supplementary Fig. 57. ^{1}H-NMR spectrum (400 MHz) of TIPS-An-BE in $CDCl_3$.

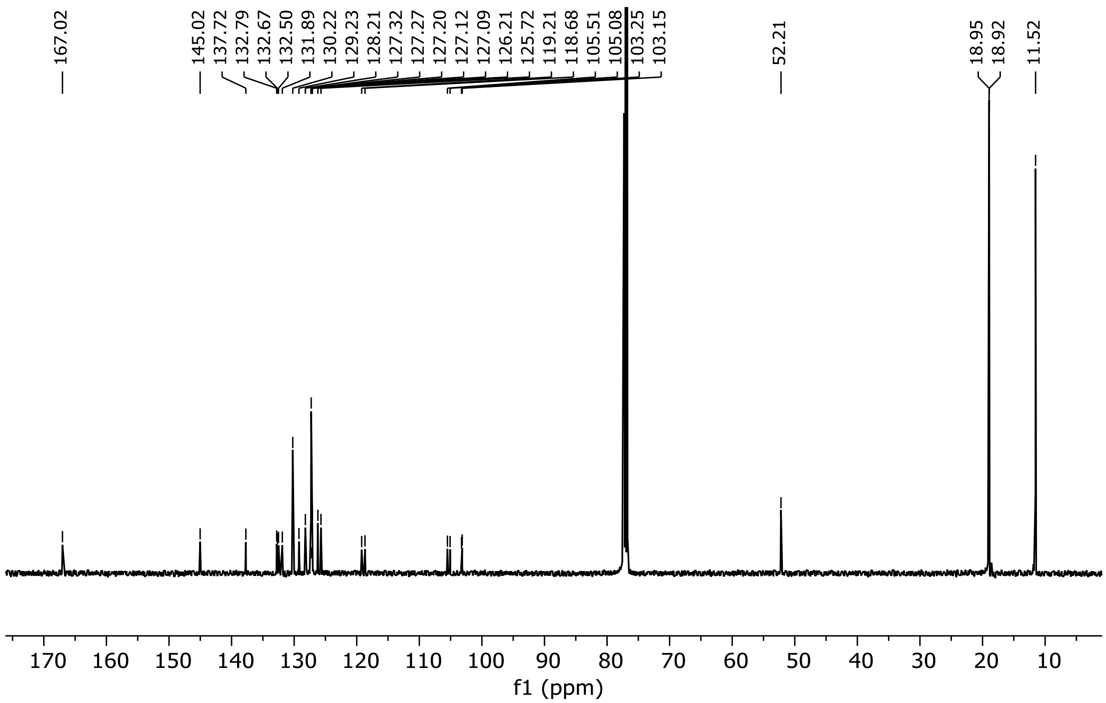

Supplementary Fig. 58. ^{13}C-NMR spectrum (126 MHz) of TIPS-An-BE in $CDCl_3$.

IV. Supplementary References

1. Plimpton, S. Fast Parallel Algorithms for Short-Range Molecular Dynamics. *J. Comput. Phys.* **117**, 1–19 (1995).
2. García-Gallego, S., Hult, D., Olsson, J. V. & Malkoch, M. Fluoride-Promoted Esterification with Imidazolide-Activated Compounds: A Modular and Sustainable Approach to Dendrimers. *Angew. Chem. Int. Ed.* **54**, 2416–2419 (2015).
